# Pharmacological characterization of the seven human NOX isoforms and their inhibitors

**DOI:** 10.1016/j.redox.2019.101272

**Published:** 2019-07-11

**Authors:** Fiona Augsburger, Aleksandra Filippova, Delphine Rasti, Tamara Seredenina, Magdalena Lam, Ghassan Maghzal, Zahia Mahiout, Pidder Jansen-Dürr, Ulla G. Knaus, James Doroshow, Roland Stocker, Karl-Heinz Krause, Vincent Jaquet

**Affiliations:** a Department of Pathology and Immunology, Centre Médical Universitaire, Geneva, Switzerland; b Institute for Biomedical Aging Research (IBA), University of Innsbruck, Innsbruck, Austria; c Conway Institute, University College Dublin, Dublin, Ireland; d National Cancer Institute, Bethesda, MD, 20816, USA; e Victor Chang Cardiac Research Institute, Vascular Biology Division, 405 Liverpool Street, Darlinghurst, NSW, 2010, Australia; f St Vincent's Clinical School, University of New South Wales, NSW, Australia

**Keywords:** NADPH oxidase, NOX, Reactive oxygen species, Small molecule inhibitors

## Abstract

**Background:**

NADPH oxidases (NOX) are a family of flavoenzymes that catalyze the formation of superoxide anion radical (O_2_^•-^) and/or hydrogen peroxide (H_2_O_2_). As major oxidant generators, NOX are associated with oxidative damage in numerous diseases and represent promising drug targets for several pathologies. Various small molecule NOX inhibitors are used in the literature, but their pharmacological characterization is often incomplete in terms of potency, specificity and mode of action.

**Experimental approach:**

We used cell lines expressing high levels of human NOX isoforms (NOX1-5, DUOX1 and 2) to detect NOX-derived O_2_^•-^ or H_2_O_2_ using a variety of specific probes. NOX inhibitory activity of diphenylene iodonium (DPI), apocynin, diapocynin, ebselen, GKT136901 and VAS2870 was tested on NOX isoforms in cellular and membrane assays. Additional assays were used to identify potential off target effects, such as antioxidant activity, interference with assays or acute cytotoxicity.

**Key results:**

Cells expressing active NOX isoforms formed O_2_^•-^, except for DUOX1 and 2, and in all cases activation of NOX isoforms was associated with the detection of extracellular H_2_O_2_. Among all molecules tested, DPI elicited dose-dependent inhibition of all isoforms in all assays, however all other molecules tested displayed interesting pharmacological characteristics, but did not meet criteria for *bona fide* NOX inhibitors.

**Conclusion:**

Our findings indicate that experimental results obtained with widely used NOX inhibitors must be carefully interpreted and highlight the challenge of developing reliable pharmacological inhibitors of these key molecular targets.

## Graphical abstract

**Figure:**
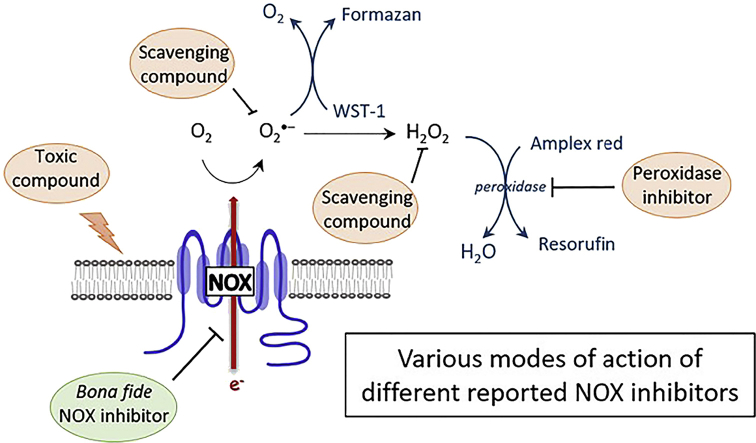

## Abbreviations

CBA: coumarin boronic acid
DMSO: dimethyl sulfoxide
DPI: diphenylene iodonium
DUOX: dual oxidase
FAD-Na_2_: Flavin adenine dinucleotide disodium salt hydrate
HRP: horseradish peroxidase
NADPH: Nicotinamide adenine dinucleotide phosphate
NOX: NADPH oxidase
PMA: phorbol 12-myristate 13-acetate
ROS: reactive oxygen species
SOD: superoxide dismutase

## 1 Introduction

NADPH oxidases (NOX) catalyze the formation of superoxide anion radical (O_2_^•-^) and the downstream generation of hydrogen peroxide (H_2_O_2_). This family of membrane spanning proteins consists of seven isoforms: NOX1-5, DUOX1 and DUOX2. When active, NOX transfer electrons across biological membranes using cytosolic NADPH as electron donor and molecular oxygen as acceptor, thereby creating extracellular O_2_^•-^ as primary product. However, H_2_O_2_ is primarily detected when NOX4, DUOX1 and DUOX2 are active, suggesting that these isoforms have the additional capacity to instantly disproportionate extracellular O_2_^•-^ into H_2_O_2_.

NOX catalytic subunits consist of six transmembrane domains for NOX1-5 and seven for DUOX1 and 2. NOX isoforms differ in their tissue distribution and expression levels. They present complex modes of action: several NOX isoforms require additional subunits for full activity. The transmembrane subunit p22^phox^ is required for activity of NOX1, NOX2, NOX3 and NOX4. Cytosolic subunits are required for NOX1, NOX2 and NOX3. For NOX5, DUOX1 and DUOX2, specific phosphorylations and binding of Ca^2+^ to intracellular EF hands cause conformational change and activation of the enzymes. Additionally, transmembrane maturation factors DUOXA1 and DUOXA2 are required for DUOX1 and DUOX2 activity [1].

The physiological functions of NOX are diverse and range from host defense (NOX2), thyroid hormone synthesis (DUOX2) to otoconia formation (NOX3) [1]. Oxidative stress and redox dysregulation are typical features of numerous diseases, and uncontrolled NOX activity represents a major source of reactive oxidants in pathological oxidative damage. Numerous studies using NOX knockout mice have shown a key contribution of specific NOX isoforms in diverse models of pathologies, e.g. NOX2 in melanoma lung metastasis [2], NOX4 in stroke [3] and diabetic complications [4], supporting the concept that NOX are promising drug targets. In the last decade, pharmaceutical companies as well as academic groups have performed several screens to identify novel drugs targeting NOX. A plethora of compounds are described as NOX inhibitors [5,6], however the actual efficacy of certain small molecules on NOX activity has been challenged [[7], [8], [9]]. It is critical for researchers and clinicians in the NOX field to use reliable NOX inhibitors. In this study, we systematically tested some of the most widely used inhibitors for their potency of mitigating NOX activity, specificity among different NOX isoforms and potential off-target effects. The compounds tested in this study included GKT136901 and VAS2870 developed by pharmaceutical companies, and ebselen, apocynin, and diapocynin, which were discovered in academic settings. The flavoprotein inhibitor diphenylene iodonium (DPI), widely used as a reference compound in NOX research was also included [10].

## 2 Materials and methods

### 2.1 Reagents

Dulbecco's Modified Eagle Medium: Nutrient Mixture F-12 (DMEM/F-12), Dulbecco׳s modified Eagle medium with 4.5 g/L glucose (DMEM), Roswell Park Memorial Institute (RPMI) 1640 with Glutamax, fetal bovine serum (FBS), penicillin, streptomycin, Dulbecco's Phosphate-Buffered Saline (DPBS), Hanks׳ buffered salt solution with CaCl_2_, MgCl_2_, glucose and sodium bicarbonate (HBSS), trypsin-EDTA (0.05%), Amplex^®^ Red and Calcein-AM were purchased from Invitrogen. (Dextran T500) was purchased from Pharmacia Fine chemicals Uppsala, Sweden. Horseradish peroxidase (HRP), flavin adenine dinucleotide disodium salt hydrate (FAD-Na_2_), phorbol 12-myristate 13-acetate (PMA), ionomycin calcium, superoxide dismutase (SOD), catalase, purified myeloperoxidase, dimethyl sulfoxide (DMSO), hydrogen peroxide 35% (H_2_O_2_), 4-(2-Hydroxyethyl)piperazine-1-ethanesulfonic acid (HEPES), ethylenedinitrilotetraacetic acid (EDTA), N,N-bis(carboxymethyl)glycine (NTA), 1,2-bis(2-aminophenoxy)ethane-N,N,N′,N'-tetraacetic acid (BAPTA), 3-*sn*-phosphatidic acid sodium salt, tetramethyl benzidine, dimethylformamide, MgCl_2_, VAS2870 (CAS 722456-31-7), ebselen (CAS 60940-34-3), diapocynin (CAS 29799-22-2) and apocynin (CAS 498-02-2) were purchased from Sigma-Aldrich. Sodium citrate tribasic dehydrate was purchased from Fluka. 2-(4-iodophenyl)-3-(4-nitrophenyl)-5-(2,4-disulfophenyl)-2H-tetrazolium monosodium salt (WST-1) (CAS 150849-52-8) was purchased from Dojindo Molecular Technologies and diphenylene iodonium chloride (DPI) (CAS 4673-26-1) from Enzo life Sciences. Ficoll-Paque™ PLUS was purchased from GE Healthcare. Coumarin boronic acid (CBA), GKT137831 (CAS 1218942-37-0) and 2,2-Diphenyl-1-picrylhydrazyl (DPPH) were purchased from Cayman Chemical. Nicotinamide adenine dinucleotide phosphate (NADPH) was purchased from Toronto Research Chemicals and CaCl_2_ from AppliChem. GKT136901 (CAS 955272-06-7) was obtained from Alinda Chemical Ltd, Russia and L-012 (CAS 143556-24-5) from Wako Chemicals.

### 2.2 Cell lines

The cell lines expressing NOX1-5 were previously described in detail [7]. Briefly, CHO cells were transduced with human NOX1, NOXO1, NOXA1 and p22^phox^, the PLB-985 cell line naturally expresses NOX2, p67^phox^, p47^phox^, p40^phox^ and p22^phox^ after differentiation into granulocyte-like cells with 1.25% DMSO for 72 h. A tetracycline-inducible HEK293 T-Rex cell line expresses NOX3 under the control of the tetracycline promoter, while NOXO1, NOXA1 and p22^phox^ are expressed under the control of constitutive promoters. No signal is detected before induction of NOX3 by tetracycline, which is sufficient and essential to induce O_2_^•-^ and H_2_O_2_. NOXO1 lacks an autoinhibitory region as seen in p47^phox^ and is most likely constitutively bound at the membrane through its tandem SH3 interaction with the proline rich region of p22^phox^ [11,12]. A tetracycline-inducible HEK293 T-Rex cell lines expresses human NOX4 under the control of the tetracycline promoter and endogenous p22^phox^, HEK293 cells were stably transfected with the pcDNA3.1 vector containing human NOX5. For the DUOX isoforms, HEK293 cells were transduced with either DUOX1 or DUOXA1, or DUOX2 and DUOXA2. Both DUOX1 and DUOX2 were selected by using G418 and zeocin and maintained in DMEM with 800 μg/mL G418 and 250 μg/mL zeocin. All cell lines were cultured at 37 °C in air with 5% CO_2_, CHO cells in DMEM/F-12, PLB-985 in RPMI, and HEK293 cells in DMEM containing 4.5 g/L glucose. Each culture medium was supplemented with 10% FBS, 100 units/mL of penicillin and 100 μg/mL of streptomycin.

### 2.3 Polymorphonuclear leukocytes isolation

Human polymorphonuclear leukocytes (PMN) were isolated from fresh whole blood collected from healthy volunteers as described [13]. Briefly, 32 mL blood was collected in 8 mL sodium citrate (3.8%) and blood cells sedimented for 40 min with dextran T500 (4% in NaCl 0.9%). The upper yellowish fraction containing leukocytes was collected and centrifugated at 380×*g* for 10 min. Pellets were resuspended in PBS and separated using Ficoll Plaque™ Plus (450  ×g, with no brake) for 15 min, RT. Red blood cells were lysed by addition of 5 mL H_2_O for 30 s followed by addition of 5 mL NaCl 1.8%. Remaining PMN were collected by centrifugation for 10 min at 250 ×g, washed and resuspended in HBSS, counted and kept on ice for a maximum of 30 min until they were used for oxygen consumption measurement.

### 2.4 Western blot

Cells were lysed on ice in lysis buffer, 1% Triton X-100 in 50 mM NaCl, 10 mM MgCl_2_, 1 mM EGTA and 50 mM Tris-HCl pH 7.4, supplemented with protease inhibitors (Complete™ Mini protease inhibitor cocktail, Boehringer Mannheim). After sonication, the protein concentration in the cell lysate was determined using Bio-rad Bradford protein assay. Proteins (15 μg) were separated on SDS-PAGE gels containing 10% polyacrylamide for NOX1-5 and 7.5% polyacrylamide for DUOX1 and DUOX2, and then electrotransferred to polyvinylidene difluoride membranes. After blocking with 10% non-fat dried milk diluted in PBS-Tween 0.1%, the membranes were incubated with indicated antibodies (see Table 1) at the following dilutions (NOX1, 1:1000; NOX2, 1:500; NOX4, 1:500; NOX5, 1:1000; DUOX1, 1:1000; DUOX2, 1:1000) overnight at 4 °C under agitation, washed and blocked again. HRP-labeled secondary antibodies (1:20,000) (Cappel) were used according to the type of primary antibodies with enhanced chemiluminescence reagents (ECL reagent, Amersham Biosciences) to detect antibody binding. Molecular weights were determined by interpolation of the standard curve generated using the BioRad Precision Plus Dual Stained Color Standard.

**Table 1:** Antibodies used in for NOX detection by Western blot in NOX expressing cell lines.

NOX enzyme	Type of antibody	Origin	Ref.
NOX1	Mouse monoclonal	Prof. J. Doroshow	[14]
NOX2	Mouse monoclonal	Prof D. Roos (Mo48) LSBio LS-C85347, Seattle, WA, USA	[15]
NOX4	Rabbit monoclonal	Prof. P. Jansen-Dürr,	[16]
NOX5	Rabbit polyclonal	Our laboratory	[17]
DUOX1	Rabbit polyclonal	Prof. U. Knaus	[18]
DUOX2	Rabbit polyclonal		[18]

### 2.5 Membrane purification

PLB-985 (40 dishes 150 cm^2^) were grown in suspension and differentiated by addition of 1.25% DMSO for 72 h. HEK293 cells expressing NOX5 (40 dishes 150 cm^2^) were amplified until confluence and detached using trypsin-EDTA for 5 min followed by addition of 2 vol of pre-warmed complete growth media to inactivate trypsin. Collected cells were centrifuged at 4000 ×g for 10 min. Cells were washed with PBS and centrifuged at 1500 ×g for 10 min. The supernatant was discarded and the pellet was placed at −80 °C for at least 30 min. The frozen pellet was suspended in 1.5 mL sonication buffer containing 0.1X PBS, 11% sucrose, 120 mM NaCl, 1 mM EGTA and protease inhibitors (Complete™ Mini protease inhibitor cocktail, Boehringer Mannheim) were added just before homogenization. Samples were sonicated two times for 25 s (level 7, Branson Sonifier 250). Cell debris were removed by centrifugation at 800 ×g for 10 min at 4 °C. The supernatant was laid on a sucrose gradient consisting of a bottom layer of 1 mL 40% sucrose and an upper layer of 1 mL 17% sucrose. Ultracentrifugation was performed at 150,000 ×g in SW60 rotor for 30 min at 4 °C. Following separation, the upper cytosolic fraction was discarded and the cloudy membrane fraction was collected. Protein concentration was determined with Bradford reagent using BSA as a standard. Small aliquots were kept at −80 °C.

### 2.6 Measurement of cellular O_2_^•-^ and H_2_O_2_

Several probes were used in this study in presence of cells expressing different NOX isoforms. The fluorescent probe Amplex^®^ Red activated by HRP and the luminescent ROS-Glo™ H_2_O_2_ assay from Promega were used for H_2_O_2_ detection. The luminescent probe L-012 (8-amino-5-chloro-7-phenyl-pyrido[3,4-d]pyridazine-1,4(2H,3H)dione) activated by HRP and the colorimetric probe sulfonated tetrazolium salt (WST-1) were used for O_2_^•-^ detection. Specificity of the product detection was determined by addition of 25 U/mL superoxide dismutase or 25 μg/mL catalase. The general flavoprotein inhibitor diphenylene iodonium (DPI) 10 μM was used as reference compound for NOX inhibition. Every measurement was performed at 37 °C in kinetic mode for at least 30 min immediately after NOX activation using FlexStation 3 Multi-Mode Microplate Reader or Spectramax Paradigm (Molecular Devices). L-012-derived luminescence was determined in the visible light range, and at 450 nm for the ROS-Glo™ H_2_O_2_ assay. Excitation and emission wavelengths used with Amplex^®^ Red in presence of HRP were 550 nm and 600 nm, respectively. WST-1 reduction was detected at 440 nm and a measure at 600 nm was performed to verify basal background values. Cells were collected by trypsinization for adherent cells (CHO, HEK293) or centrifugation for non-adherent cells (PLB-985), washed with HBSS, counted and suspended in HBSS at 500,000 cells/mL. Compounds were dispensed prior addition of cells. Cells were seeded in 96-well plates (either transparent for absorbance, black for fluorescence or white for luminescence), at a density of 50,000 cells per well (100 μl for all probes except 70 μl in the ROS-Glo™ H_2_O_2_ assay, see below). After 2–3 min incubation, HBSS solution containing activators as indicated and the probe was added to reach 200 μl/well with final concentration of 50 μM L-012 and 0.05 U/mL HRP, 25 μM Amplex^®^ Red and 0.05 mU/mL HRP or 0.5 mM WST-1.

NOX1 and NOX2 were activated with the protein kinase C activator phorbol 12-myristate 13-acetate (PMA) 0.1 μM in the reaction mixture, and NOX5, DUOX1 and DUOX2 with both PMA 0.1 μM and the Ca^2+^ ionophore ionomycin 1 μM. NOX3 and NOX4 were induced by addition of tetracycline 1 μg/mL 24 h prior to the experiments as previously described [7].

The same cell density and concentration of activators were used in the ROS-Glo™ H_2_O_2_ assay, but the volumes were adjusted to reach 100 μl/well, including 20 μL of the H_2_O_2_ substrate solution prepared according to manufacturer's instructions. Plates were incubated 90 min at 37 °C in air with 5% CO_2_. Fresh ROS-Glo™ Detection Solution (100 μL/well), prepared according to manufacturer instructions, was added before the luminescence measurement.

### 2.7 LC-MS/MS determination of cellular O_2_^•-^ generation in NOX4 cells

Generation of 2-OH-E^+^ (a specific product of HE with O_2_^•-^) from hydroethidine (HE) was investigated in cells using LC/MS/MS**.** NOX4 T-rex cells were grown in 6 well plates (seeded at 1.25 × 10^5^ cells per well) for 2 days and used for determination of 2-OH-E^+^ and E^+^ at approximately 5 × 10^5^ cells per well. Induction of expression was performed by addition of tetracycline 1 μg/mL 24 h before use. On the day of the experiment, cells were washed with PBS and subsequently incubated with HE (10 μM final concentration) for 1 h, in the dark, at 37 °C. At the end of the incubation, media were removed, transferred to clean tubes and placed immediately on ice. Cells were washed and harvested in ice cold PBS. Media and the cell pellets were extracted with 200 μL of ice-cold 80% (v/v) ethanol by vigorous mixing for 1 min. Samples were then incubated on ice for 20 min before centrifugation (16,000 ×g, 20 min, 4 °C). Supernatants were then subjected to LC/MS/MS to quantify HE and selected oxidation products according to previously described method [19]. Protein content was determined by the BCA assay in separate wells on the same plate.

### 2.8 NOX2 and NOX5 activity in purified membranes

NOX2 containing membranes were used at 10 μg/mL of proteins in a reaction mixture containing 90 nM recombinant RacQ61L, 45 nM recombinant p67Np47 N chimeric protein (consisting of a fusion of the N-termini of human p67phox [residues 1–210] and p47phox [residues 1–286]), 90 μM SDS, 1 μM FAD-Na_2_ and 1 mM WST-1 or 800 μM CBA in PBS. The reaction mixture was dispensed in 96-well plates (transparent for WST-1 absorbance, black for CBA fluorescence) at 80 μL/well and the plate was incubated for 5 min at 37 °C. The reaction was initiated by addition of 20 μL/well NADPH at final concentration 100 μM. NOX5 containing membranes were used at 20 μg/mL of proteins in a reaction mixture containing 50 mM HEPES pH 7.4, 1 mM NTA, 1 mM EDTA, 1 mM BAPTA, 1 mM MgCl_2_, 5.5 μM phosphatidic acid, 1 μM FAD, 700 μM CaCl_2_ and 1 mM WST-1 or 800 μM CBA in Milli-Q H_2_O. The reaction mixture was dispensed in 96-well plates (transparent for absorbance, black for fluorescence) at 80 μL/well and 20 μL/well of NADPH at final concentration of 200 μM were added to initiate the reaction.

### 2.9 Evaluation of small molecules in membrane assays

Reported NOX inhibitors were added in 96 well plates at a single concentration as indicated to which mix containing membrane fractions and all components of the NOX2 or NOX5 membrane assays were added except NADPH. After 2–3 min incubation, 200 μM NADPH was added to start the reaction. WST-1 and CBA were used to detect O_2_^•-^ and H_2_O_2_ respectively.

### 2.10 Oxygen consumption

Oxygen consumption was determined using human neutrophils and NOX4 T-Rex HEK293 cells using the Seahorse XF24 Extracellular Flux Analyzer. Neutrophils were plated in 24-well microplates (250,000 cells/well in 200 μL HBSS) and incubated at room temperature for 15 min for attachment. The medium was changed to DMEM without sodium bicarbonate containing 10 mM Hepes prewarmed to 37 °C, and the cells were incubated for 30 min at 37 °C in a non-CO_2_ incubator. Basal oxygen consumption was recorded for 8 min followed by addition of compounds and 8 min preincubation. PMA (100 nM) was added where indicated and the oxygen consumption rate was determined for two cycles of 8 min each. The signal obtained from neutrophils without PMA treatment was subtracted as a blank, while the signal obtained from PMA + DMSO-treated neutrophils was considered as 100%.

For NOX4-dependent oxygen consumption, NOX4 T-Rex HEK cells (50,000 cells/well) were plated onto poly-l-lysine coated XF24 cell culture microplates (Seahorse), treated with 1 μg/mL tetracycline and incubated at 37 °C with 5% CO_2_ for 24 h. One hour prior to assay, the medium was changed to DMEM without carbonate buffer supplemented with 10 mM HEPES, pH 7.4 and the cells were then incubated for 1 h at 37 °C in normal atmosphere. The baseline oxygen consumption rate (OCR) was recorded 3 times before rotenone (1 μM) and antimycin A (1 μM) were added to eliminate mitochondrial oxygen consumption and 1 OCR cycle was measured. Test compounds were added in the second injection and 4 OCR cycles were recorded. Each OCR cycle was as follows: 3 min mixing; 2 min waiting; 3 min measuring. The basal OCR in tetracycline-induced cells (prior to addition of blockers of mitochondrial respiration) was considered as 100%. Data were analysed using Wave XFe 2.1.0 software. At least three independent experiments were performed for each compound.

### 2.11 Scavenging assays

Small molecules may appear as NOX inhibitors, but act as antioxidants that scavenge O_2_^•-^/H_2_O_2_, inhibit HRP in the Amplex^®^ Red assay, or are toxic to the cells. In order to test the antioxidant potential of the inhibitors, several assays were performed using serial dilutions of compounds at constant DMSO concentration: (i) 800 μM CBA [20] was incubated in presence of 10 μM H_2_O_2_ instead of cells and fluorescence measured every minute for 1 h; (ii) similarly 10 μM H_2_O_2_ was incubated in presence of 25 μM Amplex^®^ Red and 0.05 mU/mL HRP and fluorescence measured every minute for 1 h. This assay allows to detect H_2_O_2_ scavengers as well as HRP inhibitors. (iii) Another assay for determining antioxidant activity analyzes serial dilutions of compounds in the presence of free radical DPPH in methanol in transparent 96-well plates (200 μl/well). Absorbance was measured at 540 nm after 15 min incubation in the dark. All scavenging assays were performed with FlexStation 3 Multi-Mode Microplate Reader (Molecular Devices).

### 2.12 Cell viability

The effect of the compounds on cell viability was obtained using the Calcein-AM assay. Cells were prepared as for ROS measurement (see above). Cells suspensions were prepared in HBSS. Cells were preincubated at 50,000 cells/well with compounds for 10 min at room temperature before addition of 4 μM Calcein-AM. Then the plate was incubated at 37 °C for 1 h in the dark. Intracellular Calcein fluorescence was measured using Spectramax Paradigm (Molecular Devices) at 485 nm excitation and 520 nm emission.

### 2.13 Data analysis

All graphs were prepared using GraphPad Prism™ and all data are expressed as mean ± standard deviation. Sigmoidal curve-fitting of concentration-response curve, IC_50_-values and statistical tests were all performed with GraphPad Prism™. For clarity, all concentration-response curves are presented as % of control with control being the untreated condition. Superoxide equivalents were calculated from absorbance values using the Beer-Lambert Law with *ϵ*_440 *nm*_ 37×10^3^ M^−1^cm^−1^. Rates of O_2_^•-^ production were calculated from O_2_^•-^ quantity at 10 and 30 min after initiation of the reaction. Hydrogen peroxide equivalents were calculated from fluorescence values using a H_2_O_2_ standard curve. Rates of H_2_O_2_ production were calculated from the H_2_O_2_ quantity at 20 and 40 min after initiation of the reaction.

## 3 Results

### 3.1 Characterization of NOX expressing cell lines using NOX antibodies

**Fig. 1:**
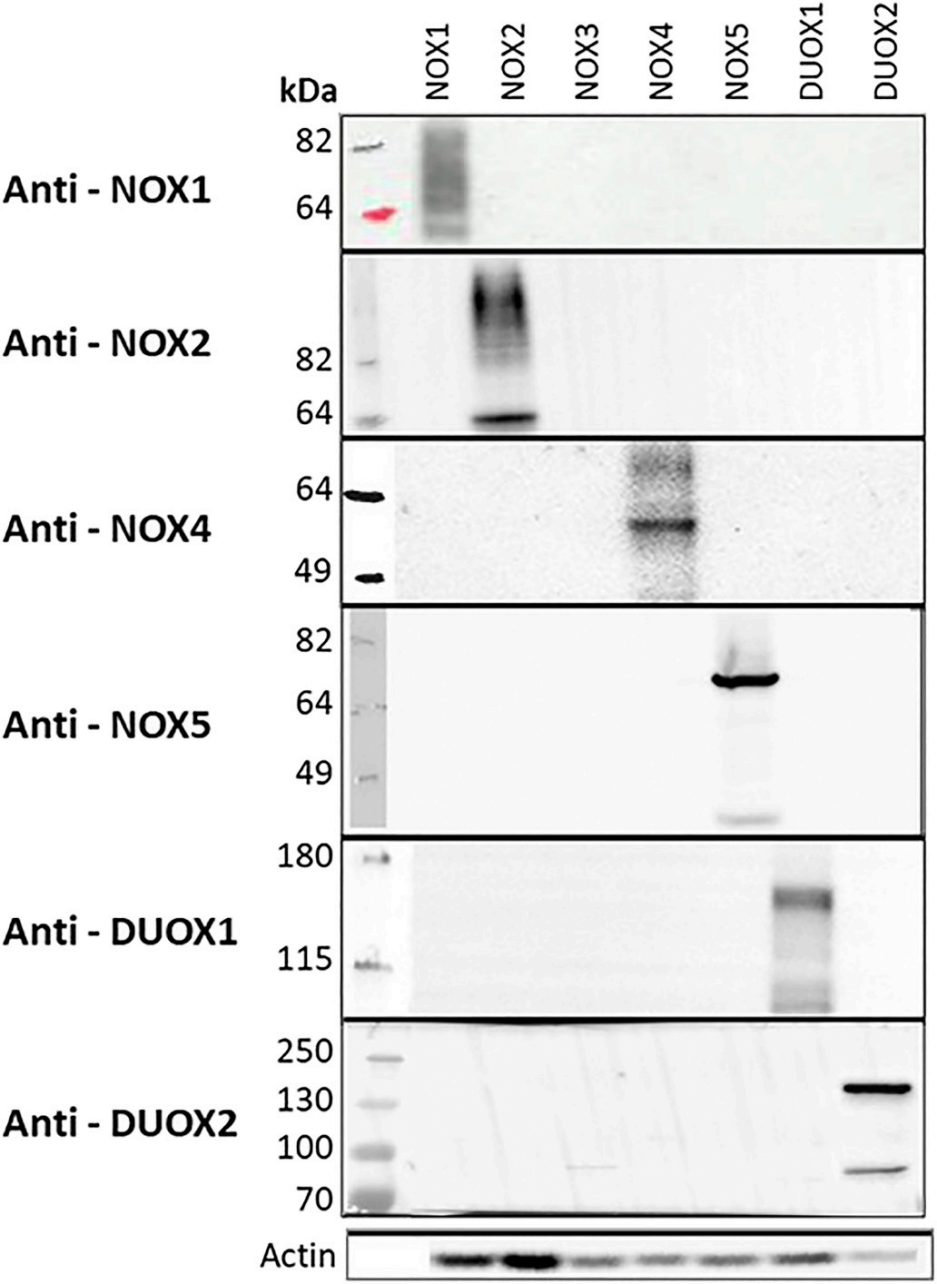
Western blot using NOX/DUOX antibodies. Each cell line (columns) was tested against NOX/DUOX antibodies (rows) by Western blot, confirming the expression of the appropriate isoform. NOX3 and NOX4 expression was induced by treatment with 1 μg tetracycline for 24 h. NOX2 expression was induced by differentiation of PLB-985 cells with DMSO 1.25% for 72 h. NOX1, NOX5 and DUOX1 and DUOX2 are constitutively overexpressed. A wide range of commercial antibodies were tested, but only the antibodies with specific reactions are presented in this study. Actin was used to show presence of proteins in the lanes. No specific antibody was found for NOX3.

The presence of NOX isoforms in the cell lines used in this study was confirmed by Western blot (Fig. 1). A mouse monoclonal NOX1 antibody detected a smeared band between 64 and 82 kDa (predicted 64.8 kDa) suggesting that human NOX1 undergoes post-translational modifications [22]. The anti-NOX2 antibody Mo48 recognizes two bands in differentiated PLB-985: one band at 64 kDa, most likely corresponding to a non-glycosylated form of NOX2 and a smeared band above 100 kDa, probably representative of glycosylated forms. The Mo48 antibody is often used for CGD diagnosis in patients’ leukocytes. However it is to our knowledge the first time that its specificity is directly compared with all other NOX isoforms [23]. No specific NOX3 antibody was found. One sharp 64 kDa specific band was observed in tetracycline-induced Trex NOX4 cells with rabbit monoclonal NOX4 antibody [16]. A NOX4 splice variant of 28 kDa has been described [24]. This band occasionally appeared in our immunoblots following NOX4 induction with tetracycline (data not shown), and most likely represents a degradation product of the full-length protein. NOX5 is detected as two bands at 70 kDa and 42 kDa, as previously described [1]. As this polyclonal antibody was raised against the EF-hand N-terminus, it was suggested that the short band represents the N terminal part of a cleaved product of NOX5 [17]. A band of approximately 170 kDa (predicted 177 kDa) is detected in DUOX1-expressing cells by a rabbit DUOX1 antibody while a band is detected at around approximately the same size (predicted 175 kDa) in DUOX2 expressing by a rabbit polyclonal DUOX2 antibody [25]. The band around 100 kDa detected by the anti-DUOX2 antibody is not specific as it appeared in non-transduced HEK293 cells (data not shown).

### 3.2 Measurements of catalytic activity of NOX expressing cells

**Fig. 2:**
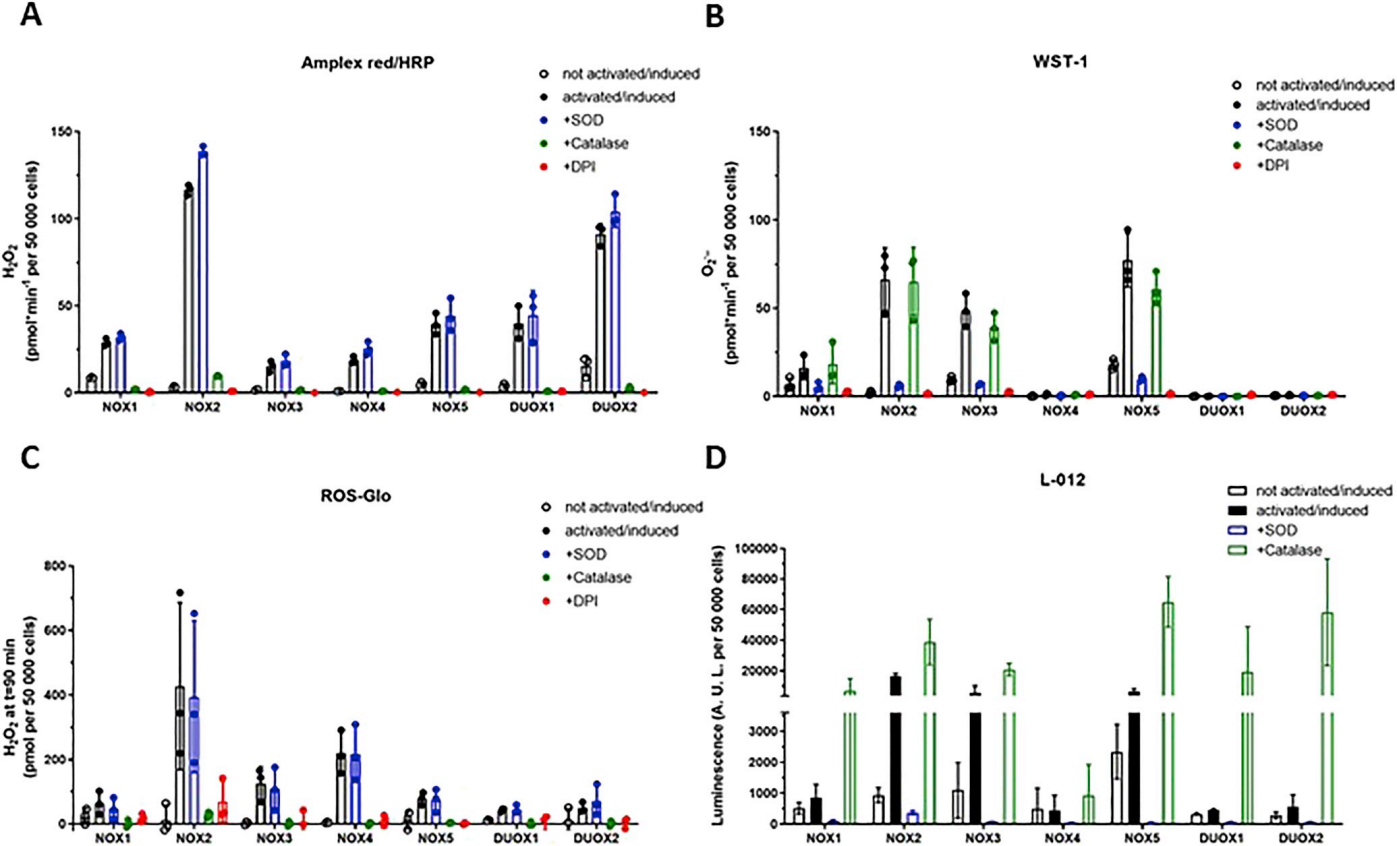
O_2_^•-^ and H_2_O_2_ generation by NOX/DUOX cell lines. NOX activity was activated/induced as described in methods. Specificity of the signal was confirmed using SOD, catalase and DPI. (A) Rate of H_2_O_2_ production measured with the Amplex^®^ Red/HRP system; (B) Rate of O_2_^•-^ measured with WST-1 probe. Data are shown in pmol per minute for 50,000 cells, calculated from a H_2_O_2_ standard curve for Amplex/red HRP and using the Beer-Lambert Law with *ϵ*_440__*nm*_ 37×10^3^ M^−1^cm^−1^ for WST-1. (C) H_2_O_2_ levels measured by ROS-Glo. Data are shown in pmol per 50,000 cells, calculated from a H_2_O_2_ standard curve; (D) Oxidant production as measured with the L-012/HRP system. Data are shown in relative unit of emitted light for 50,000 cells. Data are presented as mean ± SD of three independent experiments performed in triplicates. (For interpretation of the references to color in this figure legend, the reader is referred to the Web version of this article).

NOX activity was evaluated using several different oxidant detecting systems, including, Amplex^®^ Red, WST-1 and the chemiluminescent probes L-012 and ROS-Glo (Fig. 2).

**Supplementary figure 1:**
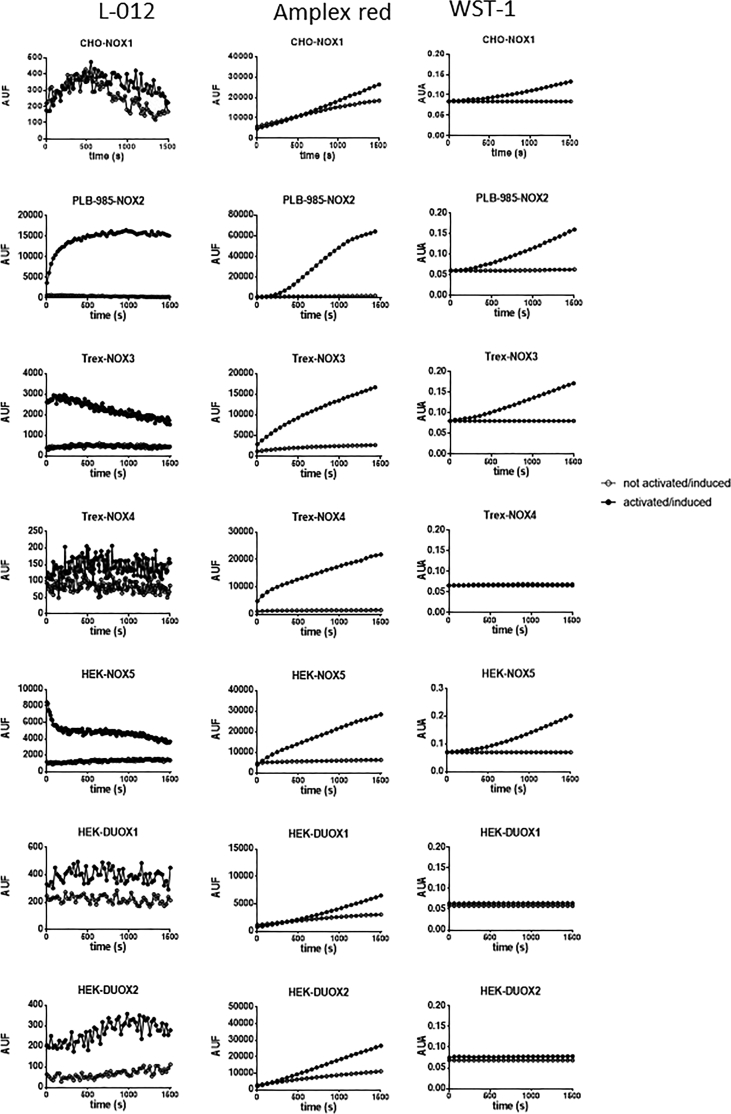
**Representative curves of the signals measured by L-012, Amplex® Red and WST-1 for each NOX-expressing cell lines.** The signal was induced by PMA 100 nM (NOX1, NOX2), induced by pretreatment with tetracycline 1 μg/mL (NOX3, NOX4) and activated with PMA 100 nM/ionomycin 1 μM (NOX5, DUOX1, DUOX2) and measured for 1,500 s.

NOX activity was observed after addition of specific stimuli: activation of NOX1 and NOX2 by addition of PMA, activation of NOX5, DUOX1, DUOX2 by addition of PMA and ionomycin and induction of expression for NOX3 and NOX4 by addition of tetracycline for 24 h. Since the cell lines are heterologously overexpressing different NOX isoforms, the relative amounts of O_2_^•-^ or H_2_O_2_ detected should not be compared between cell lines as they most likely depend on the relative expression levels of each isoform and their subunits. The only exception is PLB-985 cell line, which endogenously expresses NOX2 and all its subunits i.e. p22^phox^, p40^phox^, p47^phox^, p67^phox^ and Rac2. Representative images of the type of signal generated by each NOX isoform and probe is documented in Supplementary Fig. 1.

**Supplementary figure 2:**
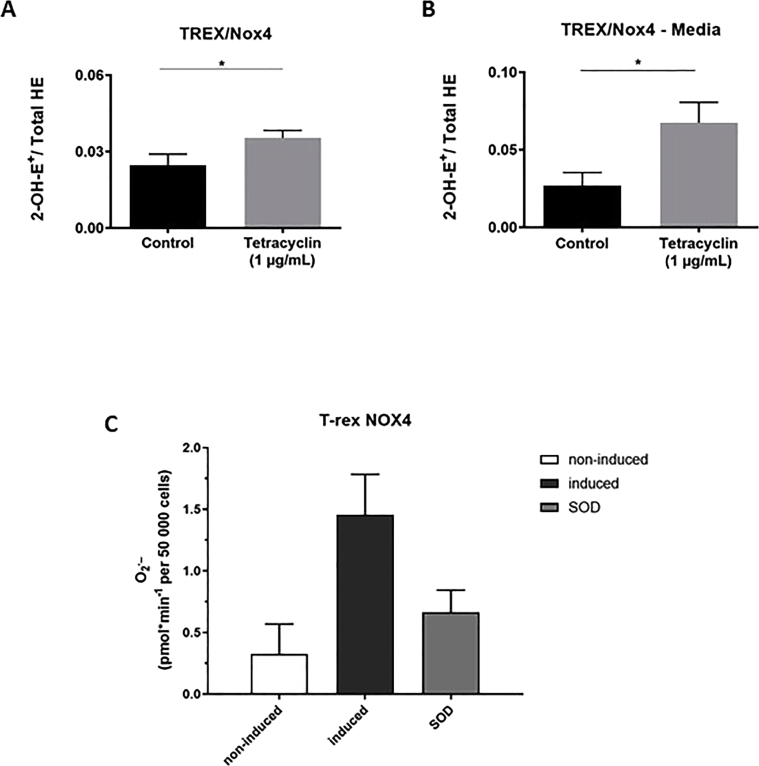
Detection of low levels of O_2_^•-^ by NOX4. NOX4 expression was induced by addition of tetracycline 1 μg/mL for 24h and hydroethidine (HE) was added for 60 min. The specific product of HE by O_2_^•-^ was measured by LC-MS-MS in cells and in the medium showing that NOX4 generates O_2_^•-^ (A), which is released in the medium (B). Detection of O_2_^•-^ by WST-1 similarly shows that NOX4 releases low levels of extracellular O_2_^•-^. Data are presented as mean ± SD of minimum three separate experiments with Mann Whitney test (ns: p>0.05; *: p≤0.05; **: p≤0.01; ***: p≤0.001). (C) Rate of O_2_^•-^ measured with WST-1 probe in cells. Data are shown in pmol per minute for 50,000 cells, calculated using the Beer-Lambert Law with *ϵ*_440 *nm*_ 37∙10^3^ M^-1^.cm^-1^.

Addition of superoxide dismutase (SOD) or catalase to the reaction allows to verify the specificity of the probes. SOD catalyzes the dismutation of O_2_^•-^ into H_2_O_2_ while catalase catalyzes the decomposition of H_2_O_2_ into H_2_O and O_2_. As SOD and catalase are membrane impermeable, they will only inhibit the extracellular oxidants. On the other hand, the ROS-Glo protocol involves cell lysis and hence measures both intracellular and extracellular H_2_O_2._ As expected, catalase completely prevents Amplex Red/HRP and Ros-Glo signals while SOD eliminates WST-1 and L-012 signals. Surprisingly, the signal of L-012 is drastically enhanced in presence of catalase (Fig. 2), while it was expected that decreasing H_2_O_2_ would decrease L-012 luminescence [26]. Addition of diphenylene iodonium (DPI) 10 μM inhibits all NOX isoforms. Although H_2_O_2_ is the primary product of NOX1-5/DUOX1-2, Amplex^®^ Red/HRP and ROS-Glo show an increased signal following activation/induction for all isoforms confirming that all activated cells expressing any of the NOX/DUOX isoforms release H_2_O_2_ into the extracellular space. WST-1 signal is increased for NOX1, NOX2, NOX3 and NOX5. It is noteworthy that the measured amount of pmol/min of O_2_^•-^ and H_2_O_2_ is strikingly similar for NOX1, NOX2, NOX3 and NOX5, while no O_2_^•-^ is detected for DUOX1 and DUOX2. Furthermore the amount of O_2_^•-^ represents approximately one tenth of oxidant generation of NOX4 activity, the rest being H_2_O_2_. Although weak, the presence of extracellular O_2_^•-^ for cells expressing active NOX4 was confirmed by LC-MS/MS detection of the specific superoxide product of hydroethidine, i.e., 2-hydroxyethidine (Supplementary Fig. 2). ROS-Glo generates a strong signal for NOX4, which is consistent with most H_2_O_2_ being generated intracellularly in TrexNOX4 cells. Amplex^®^ Red/HRP and WST1 showed consistent signals in terms of oxidant specificity along with high signal-to-noise ratio and low background. For this reason, Amplex^®^ Red/HRP and WST1 were further used to test NOX inhibitors.

### 3.3 Evaluation of inhibitors

**Fig. 3:**
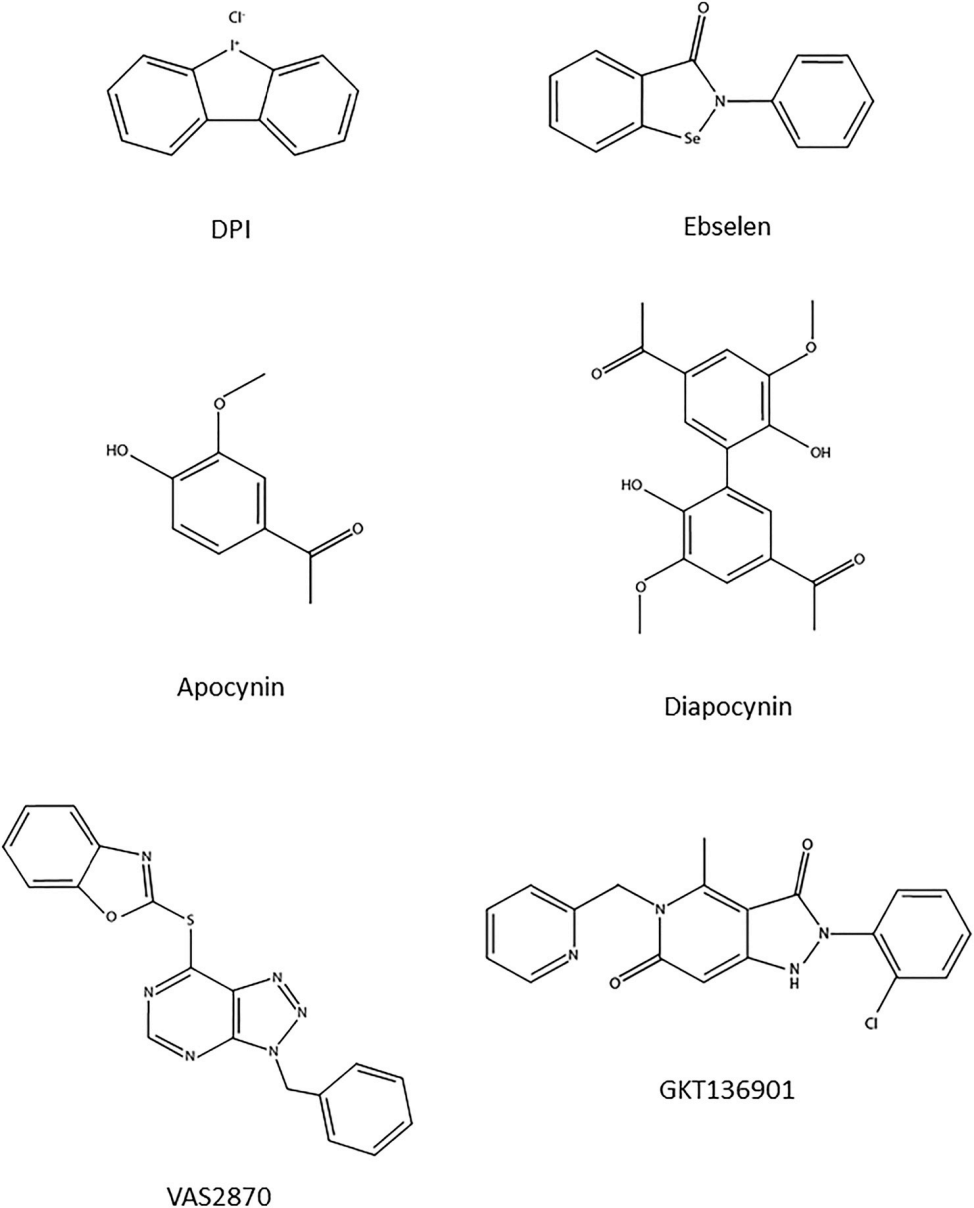
Chemical structure of reported NOX inhibitors used in this study.

**Fig. 4:**
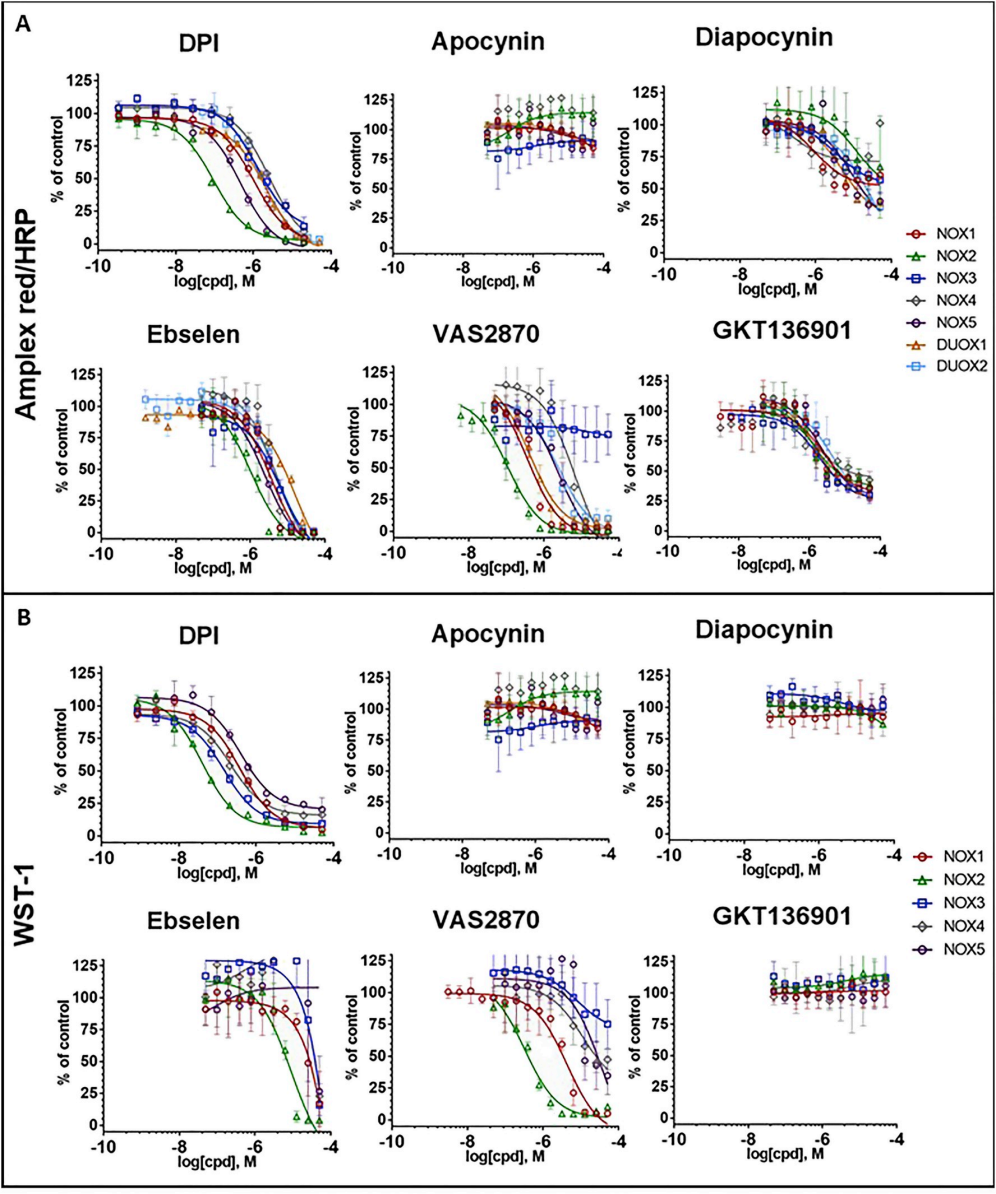
**Concentration-response of reported NOX inhibitors in NOX-dependent assays.** (A) The inhibitory activity of serial dilutions of compounds was measured on NOX expressing cells using the Amplex^®^ Red/HRP (A) and the WST-1 assays (B). Data were normalized to control (DMSO) and fitted to a sigmoidal dose-response curve using GraphPad. Calculated IC_50_ values are reported in Table 2. Data are presented as mean ± SD of n = 3 separate experiments. (For interpretation of the references to color in this figure legend, the reader is referred to the Web version of this article.)

The chemical structures of the small molecules NOX inhibitors used in this study are depicted in Fig. 3. Different concentrations of the compounds were added to NOX-expressing cells before measuring H_2_O_2_ and O_2_^•-^ using Amplex Red/HRP and WST1 (Fig. 4). As a vehicle control, constant concentration of DMSO (0.1%) was present in all dilutions. The inhibitory activity of each molecule was evaluated by calculation of IC_50_ for each probe (Table 2). IC_50_ calculations were similar between the two tests for DPI (submicromolar range) and apocynin (no inhibition), but showed lower values in Amplex^®^ Red/HRP system for all other compounds. Potential interference of the compounds with the assays used in this study were evaluated in additional cell free assays.

**Table 2:** IC_50_ (μM) of each compound tested for each NOX isoform using Amplex red/HRP and WST-1.

Name	NOX1 (CHO)		NOX2 (PLB)		NOX3 (T-REx)		NOX4 (T-REx)		NOX5 (HEK)		DUOX1 (HEK)	DUOX2 (HEK)
	AR	WST-1	AR	WST-1	AR	WST-1	AR	WST-1	AR	WST-1	AR	AR
DPI	0.9	0.4	0.1	0.1	2.2	0.2	2.7	0.4	0.4	1.2	1.4	1.7
Ebselen	2.9	31.6	1.1	6.5	2.9	35	4.1	23.7	2.1	10.1	4.2	4.2
Apocynin	>50	>50	>50	>50	>50	>50	>50	>50	>50	>50	>50	>50
Diapocynin	32.7	>50	38.9	>50	>50	>50	>50	>50	16.6	>50	13.6	21.7
VAS2870	0.5	2.8	0.1	0.4	>50	>50	6.2	24.7	2.1	28.5	0.7	2.7
GKT136901	9.2	>50	12.2	>50	5.7	>50	2.7	>50	8	>50	5.9	9.8

**Fig. 5:**
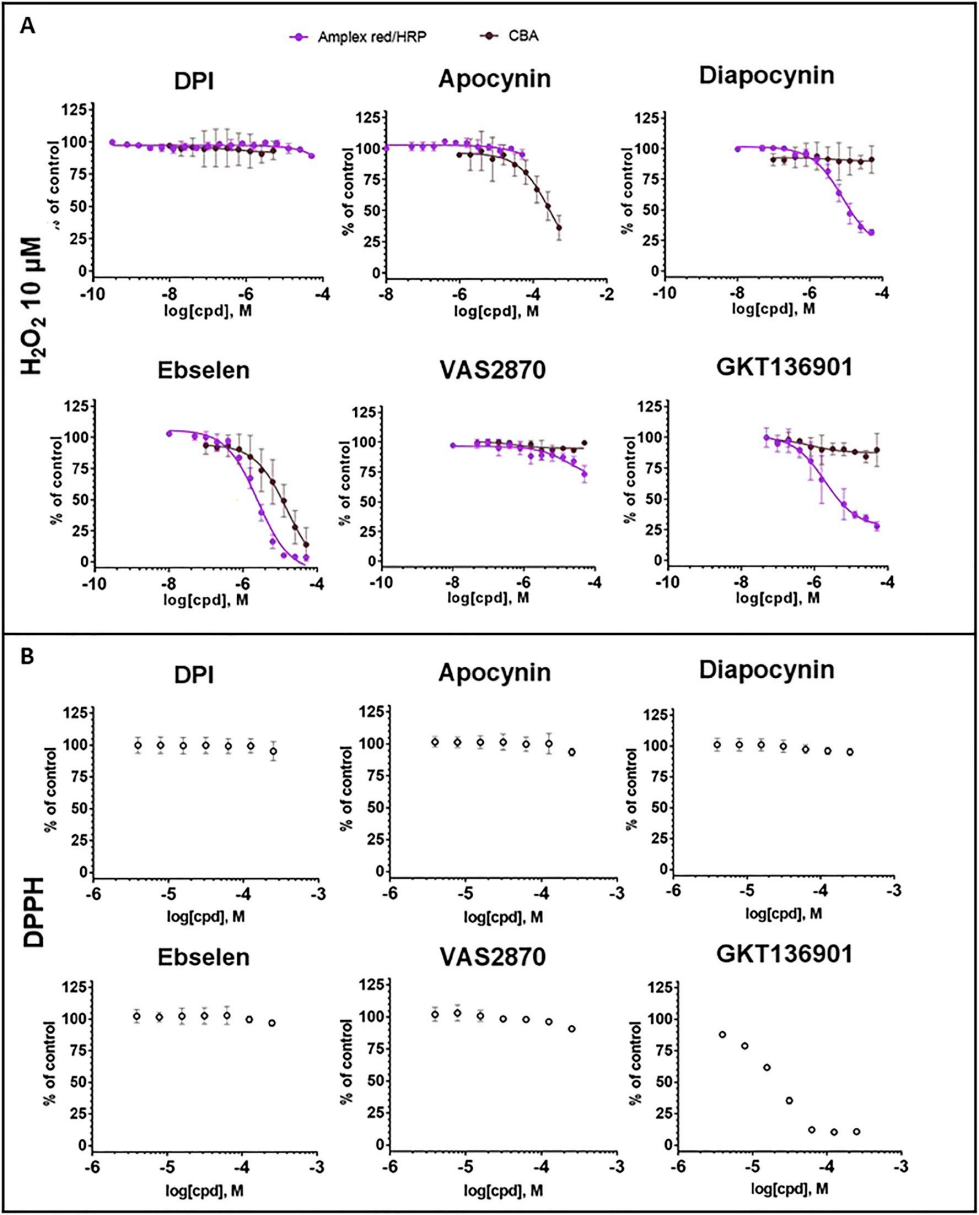
**Concentration-response of reported NOX inhibitors in NOX-independent assays** Antioxidant capacity of compounds in the absence of cell-derived NOX was evaluated using (A) H_2_O_2_ (10 μM) using CBA (brown) and Amplex^®^ Red/HRP (purple) and (B) using direct scavenging of the free radical DPPH. Data were normalized to control (DMSO) and fitted to a sigmoidal dose-response curve using GraphPad. Calculated IC_50_ values are reported in Table 3. Data are presented as mean of three independent experiment ± SD. (For interpretation of the references to color in this figure legend, the reader is referred to the Web version of this article.)

**Supplementary figure 3:**
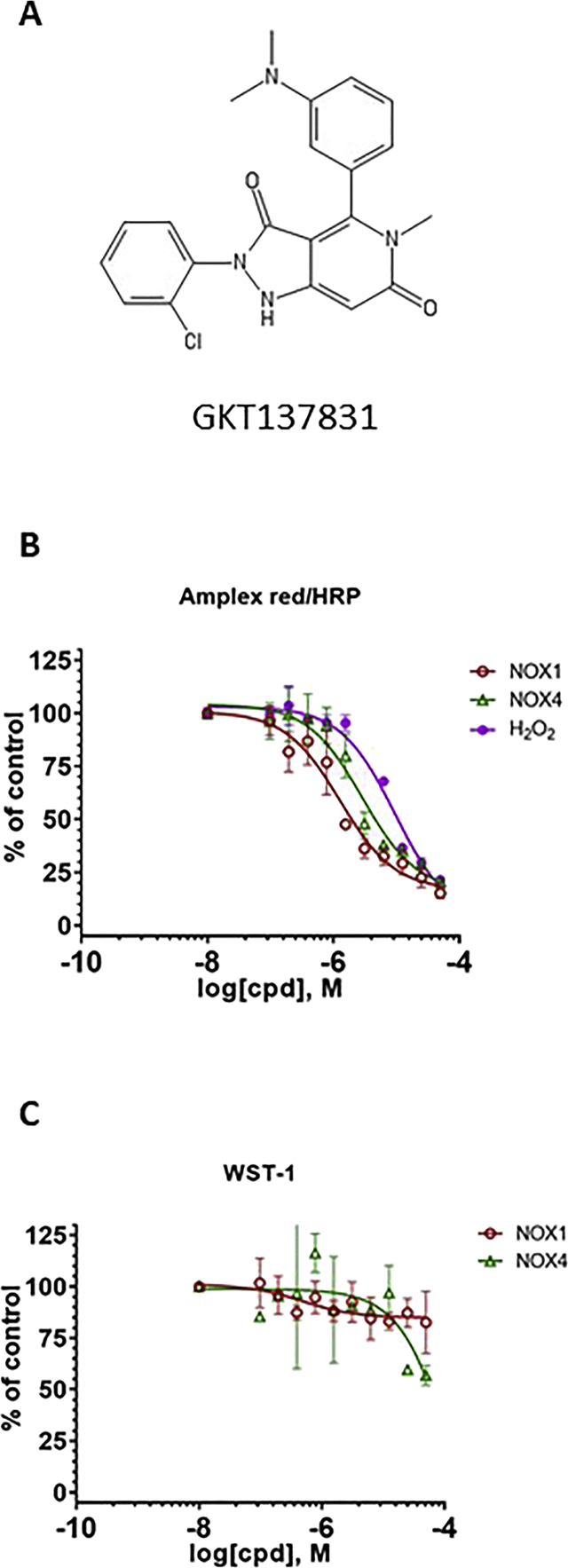
**Concentration-response ofGKT13781in NOX1 and NOX4 expressing cells.** (A) Chemical structure of GKT137831. The inhibitory activity of GKT137831 was measured using Amplex red/HRP (B) and WST-1 (C). GKT137831 inhibits the Amplex red/HRP signal even in the presence of H_2_O_2_ alone and is inactive on both NOX1 and NOX4 in the WST-1 assay. Data were normalized to control (DMSO) and fitted to a sigmoidal dose-response curve using GraphPad. Data are presented as mean of three experiments ± SD.

First the Amplex^®^ Red/HRP and CBA reaction assay was performed with 10 μM H_2_O_2_ in the presence of different concentration of compounds in cell-free systems (Fig. 5). These simple assays indicated that ebselen, diapocynin and GKT136901 inhibited the Amplex^®^ Red/HRP signal, confirming their interference with this assay and explaining over-estimation of IC_50_ for these compounds compared to the WST-1 assay. Note that the close analogue of GKT136901, GKT137831 displays a similar profile (Supplementary Fig. 3). Only Ebselen and apocynin (at high concentration) inhibited the CBA signal. As CBA oxidation is independent of a peroxidase, this suggests a direct scavenging of H_2_O_2_. Secondly, the observed direct reaction with DPPH indicated the general reducing activity of GKT136901. Elimination of the DPPH radical signal is a selectivity assay for reducing agents that often also react with compound I formed by heme-containing peroxidases like HRP (added to the Amplex^®^ Red assay) as they engage in metabolism of H_2_O_2_. Finally, to assess toxicity as a cause of signal decrease in enzyme activity measurements, cell viability measurements were made (Table 3). These indicated that part of the apparent inhibition of NOX activity by ebselen and VAS280 may in fact be due to cell toxicity.

**Table 3:** Off target effects of tested compounds. Cytoxicity is expressed in LC_50_ (μM) and scavenging effect in IC_50_ (μM).

Name	Calcein-AM			AR	Absorbance
	CHO	HEK/T-REx	PLB	H_2_O_2_	DPPH
DPI	>50	>50	>50	>50	>50
Ebselen	3.3	1.8	3.1	3.3	>50
Apocynin	>50	>50	>50	>50	>50
Diapocynin	>50	>50	>50	11.5	>50
VAS2870	2.5	4.2	4.4	>50	>50
GKT136901	>50	>50	>50	1.9	21.7

**Fig. 6:**
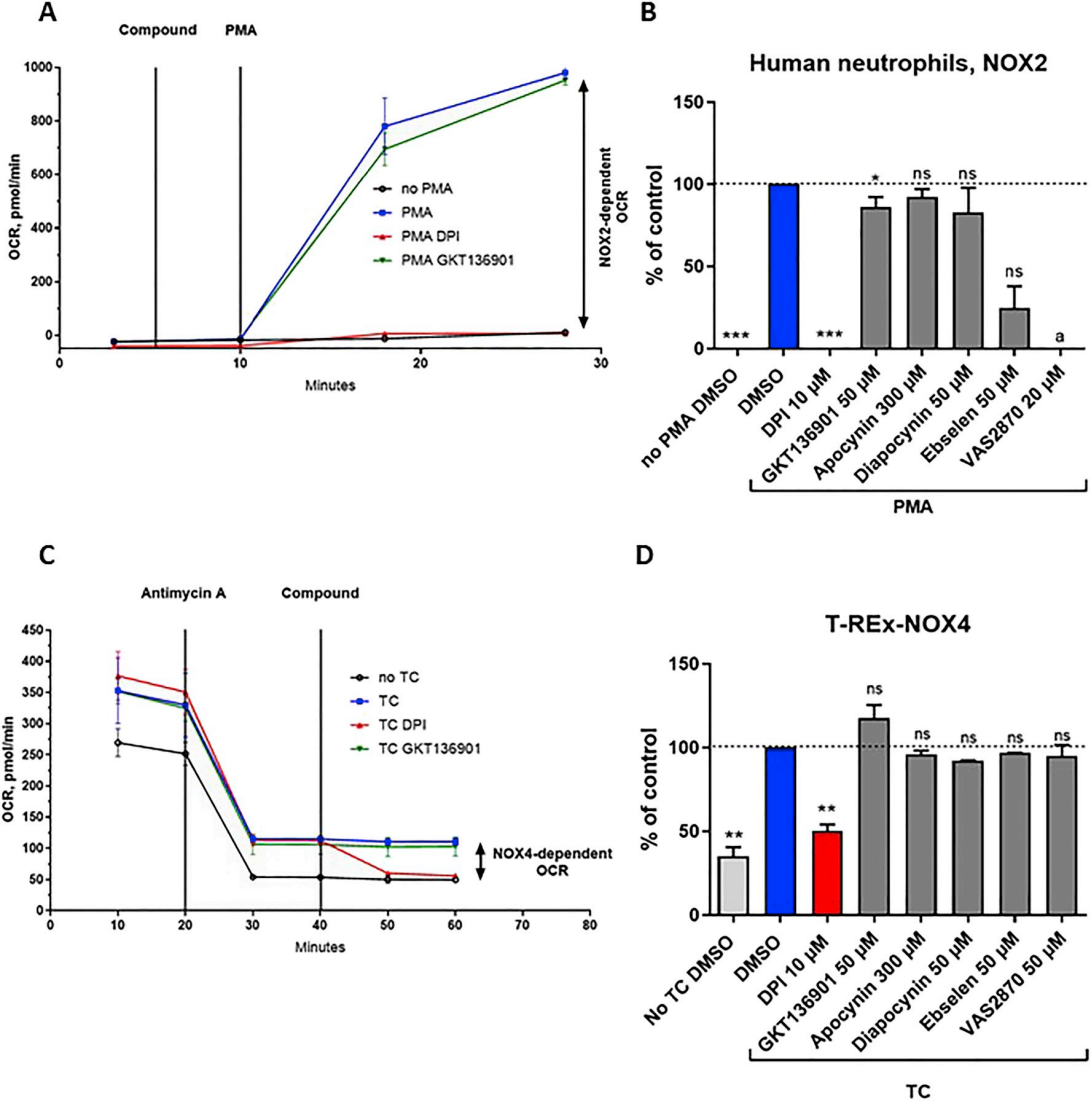
**Oxygen consumption in human neutrophils and tetracycline induced NOX4-expressing cells.** (A) Representative pattern of oxygen consumption by human neutrophils. Each point of the curve represents the mean of quadruplicates ± SD (B) Percent of inhibition of NOX2-dependent OC by the compounds used in this study. (C) Representative pattern of oxygen consumption by NOX4-expressing HEK293 T-rex cells at basal level and induced by tetracycline (TC). Each point of the curve represents the mean of quadruplicates ± SD. (D) Percent of inhibition of NOX-dependent OC by the compounds used in this study. Data are presented as mean ± SD of minimum three separate experiments and Wilcoxon tests are shown vs. DMSO (ns: p > 0.05; *: p ≤ 0.05; **: p ≤ 0.01; ***: p ≤ 0.001). ^a^Indicates that interference of the tested compound with the reagent was observed (cytotoxicity) potentially obscuring a real pharmacological effect.

Additional NOX functional assays were performed to further characterize the compounds. WST-1 and Amplex^®^ Red/HRP measure the products formed by NOX, namely O_2_^•-^ and H_2_O_2_. An alternative method to measure an enzymatic activity is to measure the disappearance of the substrate, i.e., NADPH or O_2_ in the case of NOX. We used the Seahorse™ to measure O_2_ consumption (OC) by human neutrophils and T-rex NOX4 cells (Fig. 6). Neutrophils have a very low mitochondrial OC; NOX2 activation by addition of 100 nM PMA induced strong NOX2-dependent OC (up to 900 pmol/min), which was inhibited by DPI, ebselen and VAS2870 (cytotoxic), but not by GKT136901 (50 μM) and only weakly by apocynin (at 300 μM) or diapocynin (50 μM). On the other hand, NOX4 T-rex cells have a high mitochondrial OC (around 300 pmol/min); NOX4 induction by addition of 1 μg/mL tetracycline for 24 h induced an increase of basal OC to 350 pmol/min. The mitochondrial component of OC was inhibited by antimycin A (1 μM). Remaining non-mitochondrial OC which was inhibited by DPI (10 μM) is considered to be due to NOX4 (approximately 50 pmol/min). Interestingly, the OC data for NOX2 showed a pattern similar to WST-1 measurements: only DPI, VAS2870, and ebselen inhibited NOX2, while only DPI inhibited NOX4-dependent OC.

**Fig. 7:**
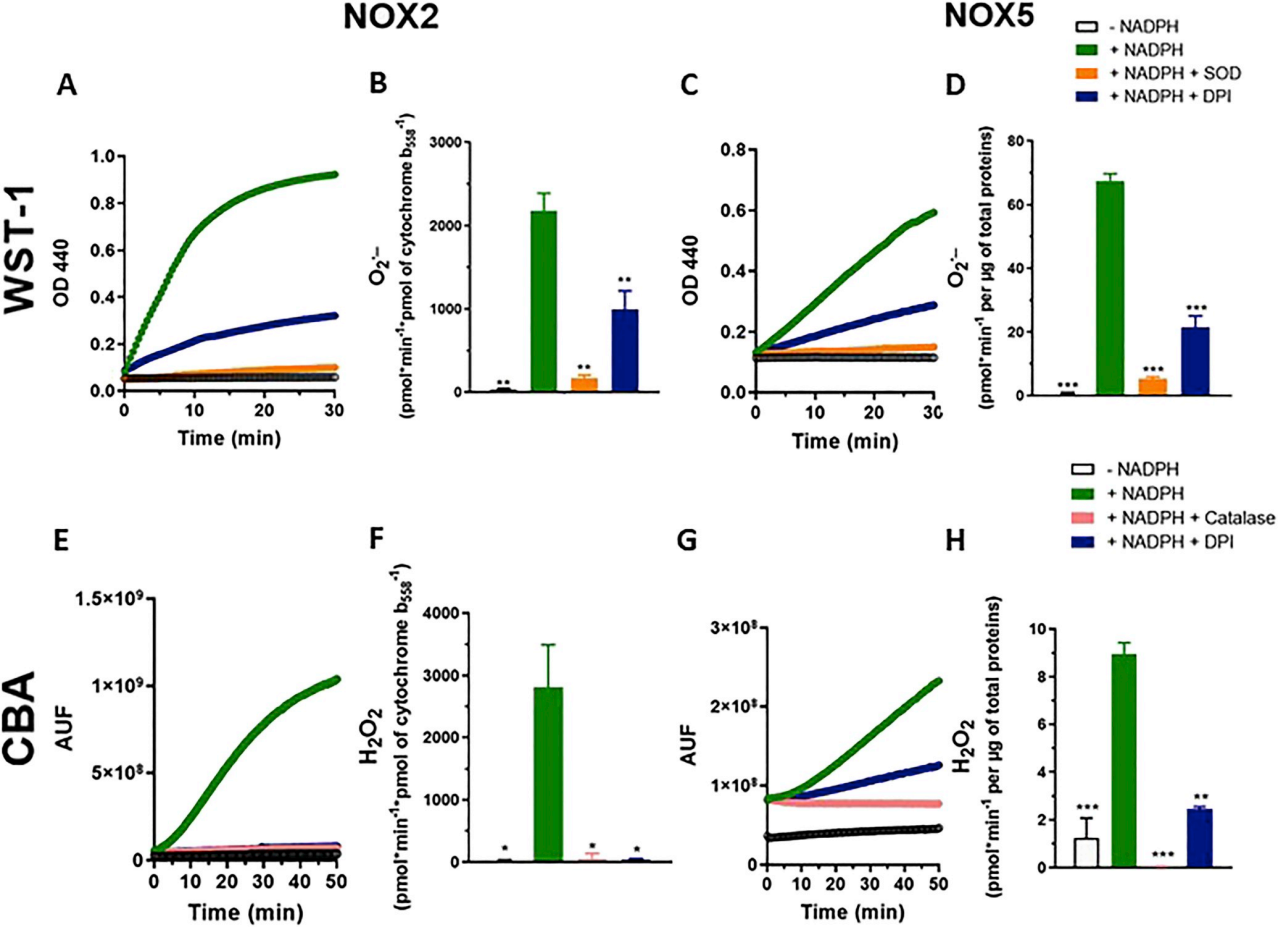
O_2_^•-^ and H_2_O_2_ generation by NOX2 and NOX5 membrane fractions: Representative curves and quantification of the rate of O_2_^•-^ and H_2_O_2_ production obtained with membrane particulate fractions using a reconstituted NOX2 system comprising purified PLB-985 membranes and recombinant Rac GTPase and a p47-p67 chimeric protein (A, E), and in the NOX5 system comprising purified membranes of HEK293 cells overexpressing NOX5 in controlled high Ca^2+^ (700 μM) (C, G) using WST-1 (A,C) and CBA (E; G). In both systems and with both probes reaction was initiated by addition of NADPH. Specificity of the signal was confirmed using DPI, SOD or catalase. Quantification of the rate of O_2_^•-^ (B, D) and H_2_O_2_ production (F, H). Oxidant formation is expressed in pmol per min per pmol of cytochrome b_558_ calculated for the NOX2 system as described [21]. As NOX5 concentration was not measurable in membrane fractions, oxidant formation is expressed in pmol per min per total amount of membrane proteins. Data are presented as mean ± SD of n = 3 separate experiments and Welch's tests are shown vs. activated membranes by NADPH (ns: p > 0.05; *: p ≤ 0.05; **: p ≤ 0.01; ***: p ≤ 0.001).

**Fig. 8:**
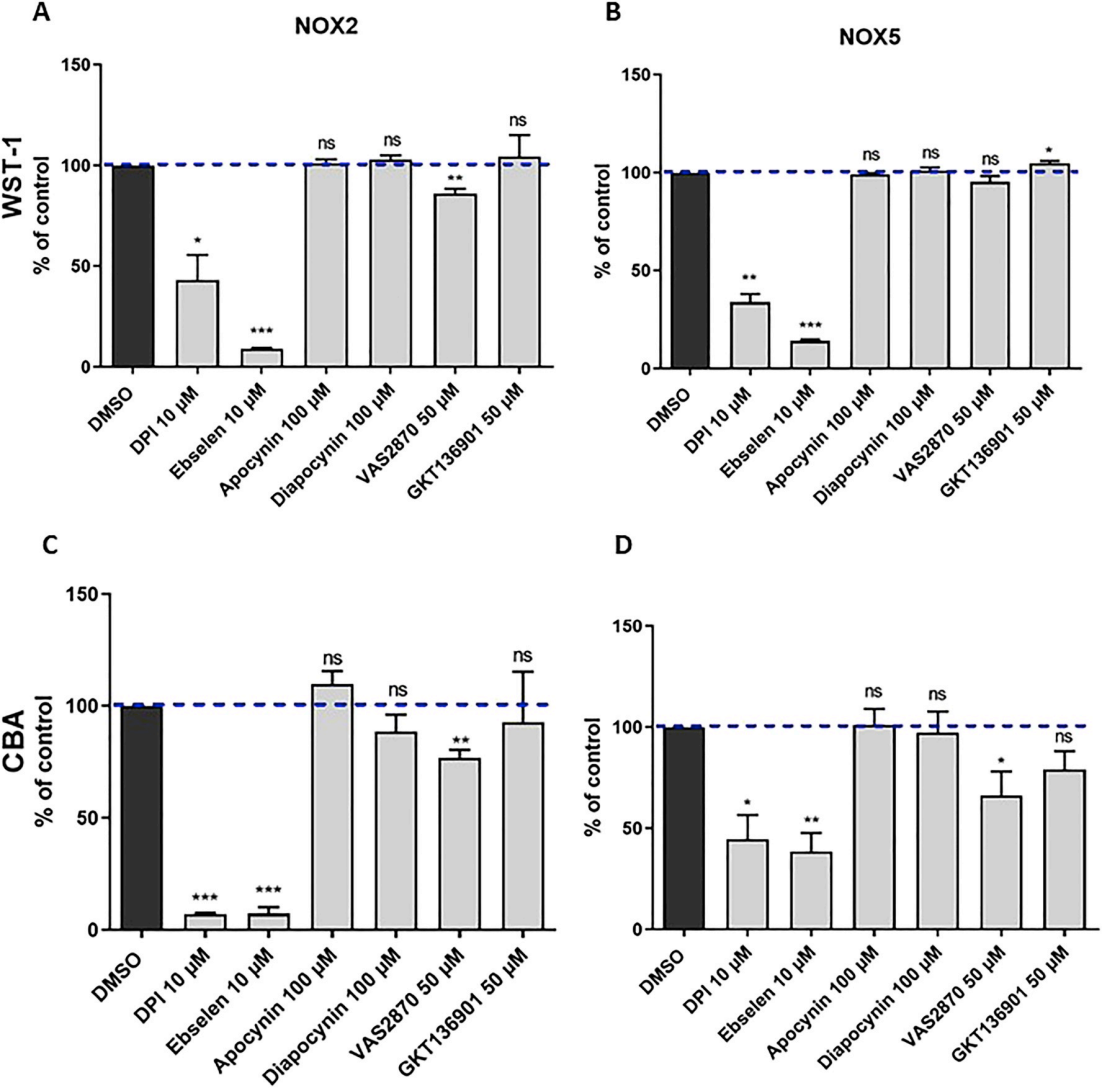
Impact of reported NOX inhibitors on O_2_^•-^ and H_2_O_2_ generation in NOX2 and NOX5 membrane fractions: The inhibitory activity of compounds at indicated concentrations was measured on NOX2 membranes (A, C) and NOX5 membranes (B, D) using WST-1 (A, B) and CBA (C, D). Data are presented as mean ± SD of minimum three separate experiments and Welch's tests are shown vs. DMSO (ns: p > 0.05; *: p ≤ 0.05; **: p ≤ 0.01; ***: p ≤ 0.001).

**Supplementary figure 4:**
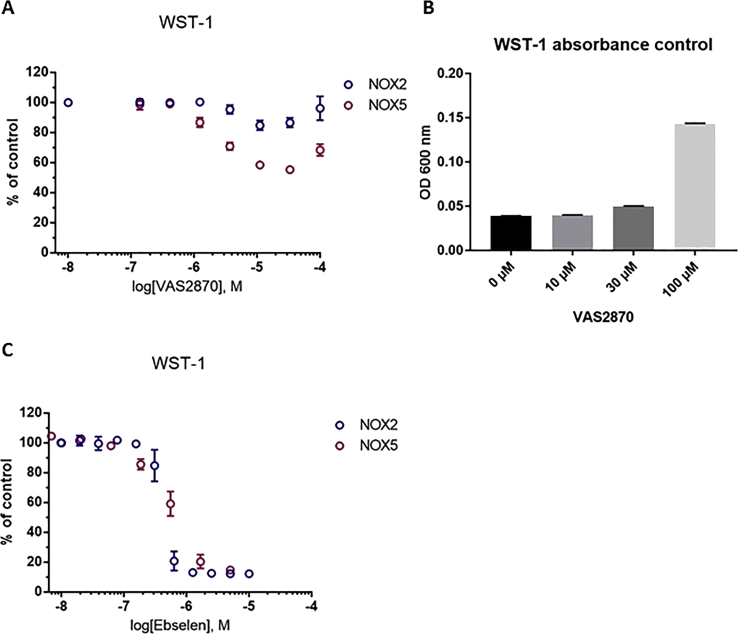
**Concentration-dependent response of VAS2870 and Ebselen in NOX2 and NOX5 membranes.** The inhibitory activity of VAS2870 (A) and Ebselen (C) was measured on NOX2 membranes and NOX5 membranes using WST-1. Precipitation of VAS2870 was visible as an increase of absorbance at 600 nm in the measured wells with the highest concentration of compound (B).

As the activity of several NOX isoforms depends on kinases or increases in cellular Ca^2+^, small molecules inhibiting these upstream pathways may appear as NOX inhibitors, whereas a *bona fide* small molecule NOX inhibitor should interact directly with its target. In order to test a direct inhibitory effect of compounds, cell free assays using cell membrane fractions containing NOX2 and NOX5 were performed. Fig. 7 shows representative curves obtained with WST-1 and CBA as well as the rate of O_2_^•-^ and H_2_O_2_ production. WST-1-dependent signals were inhibited by SOD while CBA-dependent signals was decreased by catalase, confirming the specificity of these probes for O_2_^•-^ and H_2_O_2_ respectively. Only a fraction of the WST-1 signal was inhibited by DPI suggesting a possible NOX-independent oxidant generation in this assay. Ebselen (50 μM) inhibited both NOX2 and NOX5-dependent signals, and in a concentration-dependent way while VAS280 showed some level of inhibition (Supplementary Fig. 4). As VAS2870 has low solubility in aqueous solutions (ALOGPS = 2.90 as calculated by http://www.vcclab.org), this may indicate that the compound precipitated at higher concentrations and lost its inhibitory activity. All other compounds were inactive in both assays (Fig. 8).

## 4 Discussion

In this study, we present for the first time a direct comparison of the O_2_^•-^ and H_2_O_2_ generation for all seven isoforms of the human NOX family proteins using a panel of assays designed for the validation of NOX antibodies and small molecules NOX inhibitors commonly used in the literature. We performed a variety of assays for the pharmacological characterization of small molecules NOX inhibitors to measure (i) NOX inhibitory potency, (ii) NOX isoform specificity, and (iii) off-target effects including cytotoxicity and potential oxidant scavenging activity. For evaluating inhibitor efficacy and specificity we used stable cell lines, each expressing a single NOX isoform (and the required components for this NOX isoform) as confirmed by immunoblots of cell extracts. We tested various antibodies to validate the NOX expressing cell lines and identified specific antibodies able to recognize overexpressed NOX by Western blot for all NOX isoforms except for NOX3.

To measure the efficacy of NOX inhibitors, trustworthy systems have to be used. We confirmed the specificity of the type of oxidant by addition of the enzymatic scavengers catalase and SOD, confirming that Amplex^®^ Red/HRP and Ros-Glo allows measurement of H_2_O_2_ and WST-1 allows measurement of O_2_^•-^. The situation was more complex with L-012: upon activation/induction of NOX, L-012 alone does not generate light (data not shown). With the exception of PLB-985, which contain an endogenous peroxidase (i.e. MPO), chemiluminescence of L-012 requires addition of HRP (data not shown), suggesting that L-012 serves as substrate for HRP in a H_2_O_2_-dependent way. Intriguingly, biochemiluminescence was abolished by addition of SOD, suggesting that extracellular O_2_^•-^ is responsible for the signal, while addition of catalase greatly enhanced luminescence rather than decreasing it. Zielonka et al [26] have recently analysed the chemistry of the L-012 reaction and have shown that, in spite of the fact that the reaction is inhibited by SOD, L-012 in fact measures NOX-derived H_2_O_2_ in presence of a peroxidase -this point is corroborated by the fact that L-012 detects DUOX activity. Indeed, in presence of O_2_, O_2_^•-^ is self-generated during peroxidation of L-012 by H_2_O_2_ and a peroxidase. This chemically generated O_2_^•-^ in turn oxidizes L-012 to generate light. As catalase transforms H_2_O_2_ into H_2_O and O_2_, it is therefore possible that this increase of O_2_ concentration intensifies the self-generation of O_2_^•-^ and thus massively enhances the chemiluminescent signal. Another interpretation is that catalase/H_2_O_2_ is more effective in oxidizing L-012 to its radical than HRP/H_2_O_2._ Although the L-012/HRP chemiluminescent system is quite sensitive and detects NOX activity in presence of a peroxidase for all NOX isoforms, low signals obtained for some isoforms (NOX1 and NOX4) as well as its complicated chemistry pleads against its use to test small molecule NOX inhibitors. Another limitation resides in the fact that the amount of oxidant cannot be quantified using this probe. Altogether, although L-012 is a sensitive probe which can detect NOX activity, L-012 data should be carefully evaluated. As expected, NOX1, 2, 3 and 5 generated comparable amounts of extracellular O_2_^•-^ and H_2_O_2_ in cells while the activity of DUOX1 and DUOX2 was only detected with H_2_O_2_-dependent probes [27]. In the case of NOX4, about 10 times more H_2_O_2_ was detected compared with O_2_^•-^. This observation is consistent with a previous report [28], but we confirmed that in the presence of active NOX4 small amounts of O_2_^•-^ are formed. Interestingly oxygen consumption measurement of NOX4 indicated a good correlation between the amount of consumed oxygen and H_2_O_2_ production. However, even in condition of overexpression, oxygen consumption by NOX4 is minor compared to mitochondrial oxygen consumption and that associated with the NOX2-dependent oxidative burst.

Characterization of reported NOX inhibitors was performed using the following flowchart: 1) IC_50_ values were calculated for extracellular H_2_O_2_ (Amplex^®^ Red/HRP) and O_2_^•-^ (WST-1) using NOX expressing cell lines; 2) concentration-dependent values were obtained for all off-target assays including cytotoxicity, Amplex^®^ Red/HRP interference, H_2_O_2_ scavenging using CBA and radical scavenging activity using the DPPH assay; 3) compounds were tested at single concentration for NOX2 and NOX4 oxygen consumption assays and NOX2 and NOX5 membrane assays.

Among the six molecules tested, DPI appears as the most potent and reliable NOX inhibitor. IC_50_ obtained for DPI in both H_2_O_2_-detecting Amplex^®^ Red/HRP assay and the O_2_^•-^-detecting WST1 assay were consistent for all NOX isoforms (sub-micromolar to low micromolar range). Neither toxicity, nor off-target effects could explain the NOX inhibitory effect. DPI potently inhibited NOX2 and NOX4-dependent oxygen consumption and oxidant generation in NOX2 and NOX5 membrane assays. Therefore, DPI shows most of the expected characteristics for a *bona fide* NOX inhibitor *in vitro*. However, DPI has several drawbacks hampering its therapeutic development. It is a flavoprotein inhibitor, which potently blocks CYP450 reductase [29], NO synthase [30] and the mitochondrial electron chain [31]. In terms of mode of action, it acts by accepting an electron from FAD, creating a radical and finally forming a covalent and irreversible bond with FAD. Irreversible binding, lack of specificity, low solubility in aqueous solutions and toxicity *in vivo* [32] restrain the use DPI as a drug. However, for short-term incubation time *in vitro*, DPI is a useful control for NOX research. This confirms that DPI is a suitable reference compound for NOX inhibition *in vitro* [33].

Ebselen inhibited all NOXs in the Amplex^®^ Red/HRP cell assay and NOX1, NOX2 and NOX3 in the WST-1 cell assay in a concentration-dependent manner. However, ebselen failed to inhibit NOX4 and NOX5, and it displayed off-target effects in the H_2_O_2_/CBA and H_2_O_2_/Amplex^®^ Red/HRP scavenging assays [34]. In addition, ebselen was cytotoxic at low concentration, most likely obscuring a real pharmacological effect on NOX. The steep slope of the concentration-dependent inhibition in WST-1 assay is quite typical of the pleiotropic cytotoxic impact of ebselen at high concentrations. Nevertheless, ebselen potently inhibits NOX2 and NOX5 in membrane assays. Ebselen is frequently identified as a hit in pharmacological screens including a screen for NOX2 inhibitors [35], as well as other enzymatic systems [36,37]. Ebselen has a complex pharmacology, possibly due to interaction and inactivation of protein cysteine residues. Nevertheless, ebselen appears safe in humans and its efficacy has been tested in several clinical trials for several pathologies involving excessive oxidant generation, such as hearing loss [38] or vascular function in diabetic patients [39]. It is however not yet approved for clinical use. Due to the fact that ebselen has a myriad of pharmacological activities and the inhibitory impact on NOX is virtually impossible to define with certainty as its IC_50_ is too close to its LD_50_ in living cells, ebselen is not useful as a pharmacological agent to study NOX.

VAS2870 was discovered by Vasopharm GmbH in a high-throughput screen for NOX2 inhibitors [40]. VAS2870 inhibits all NOX isoforms in cellular assays except NOX3. This suggests all NOX isoforms have some common characteristic, which is not shared by NOX3. VAS2870 is more potent on NOX1-, NOX2-and potentially DUOX-containing cells with IC_50_-values in the low micromolar or sub-micromolar range in both the WST-1 and Amplex^®^ Red/HRP assays without oxidant scavenging activity or interference with the Amplex^®^ red/HRP assay or the DPPH assay. However, VAS2870 is cytotoxic at low concentrations and it precipitates at higher concentrations. Also, VAS2870 showed no inhibitory activity in the NOX2 and NOX5 membrane assays, confirming a previous report for NOX2 [41]. In terms of mode of action, it was shown that VAS2870 inhibits NOX4 through unspecific thiol alkylation [42]. If so, NOX3 activity may not depend on redox active cysteine residues unlike NOX2 [43], NOX5 [44], and DUOXes [45]. Another explanation would be that NOX3 expressing cells are more resistant to the cytotoxic impact of VAS2870. Similarly to ebselen, VAS2870 showed cytotoxicity and presented an unspecific redox mode of action, compromising its usefulness as a tool to study NOX biology.

Apocynin is a nontoxic compound extracted from plants *Apocynum cannabium* or *Picrorhiza kurroa*, known for their anti-inflammatory effects. In spite of several studies reporting that the natural compound apocynin is inactive on NOX and acts as an antioxidant [8,9], it is still frequently used as a NOX2 inhibitor. In the present study, apocynin was consistently inactive for NOX2 or any other NOX isoform, even at high concentrations (300 μM) in the NOX2-dependent oxygen consumption assay. Apocynin did not interfere with the DPPH and H_2_O_2_/Amplex^®^ Red assays, but it scavenged H_2_O_2_ at high concentrations as assessed by the CBA assay. Apocynin is bioavailable *in vivo* [46] and shows therapeutic benefit in numerous mouse models of disease [47] and even in asthmatic patients [48], but these effects cannot be attributed to NOX inhibition.

It was proposed that apocynin is a prodrug that requires activation in a peroxidase-dependent mechanism [49], in particular through dimerization into diapocynin [50]. Thus we tested diapocynin and observed that diapocynin inhibited the Amplex^®^ Red/HRP assay for all NOX isoforms while it was inactive in the in WST1 assay. Diapocynin interfered with the Amplex^®^ red assay in the absence of cells. Diapocynin was inactive in the CBA/H_2_O_2_ scavenging assay, although the inhibitor could not be tested at higher concentrations due to its lower solubility in aqueous solutions compared with apocynin. No effect was observed with diapocynin in the cytotoxicity assays or DPPH neutralization. Overall, these findings are consistent with apocynin transformation into diapocynin by myeloperoxidase, and inhibition of peroxidase-dependent oxidation of probes, such as Amplex^®^ Red or luminol –as described in early reports [49,51]. Nevertheless, diapocynin has therapeutic value as it can be administered *in vivo* and shows therapeutic properties for Duchenne muscular dystrophy [52] and Parkinson disease [53], but again in a NOX-independent way.

GKT136901 and GKT137831 have been developed by Genkyotex SA and are documented as specific dual NOX1/NOX4 inhibitors with Ki-values in the nanomolar range for NOX1, 4 and 5 and in the micromolar range for NOX2 [54]. Here we show that GKT136901 is in fact inactive as NOX inhibitor, but rather interferes with peroxidase-dependent assays. GKT136901 (and GKT137831) are non-specifically inhibiting HRP, as they show similar inhibitory activity in the presence of H_2_O_2_ alone and NOX expressing cells while GKT136901 is not scavenging H_2_O_2_ in the CBA assay. GKT136901 showed reducing activity in the DPPH assay. Lack of NOX inhibition was confirmed by the WST1 and oxygen consumption assays. The documented low Ki-values of GKT136901 (and GKT137831) are probably the result of artifactual HRP inhibition as they were measured using the Amplex^®^ Red/HRP assay. Since calculation of Ki-values implies a precise knowledge of the mode of action of an inhibitor [55], the way these values were obtained is unclear and was not documented in previous papers [56]. GKT137831 is currently going through phase 2 clinical trials in diabetic nephropathy. Overall, the observed protective effect of the compound *in vivo* for diabetic nephropathy in pre-clinical studies might not stem from the inhibition of NOX, but rather from an unspecific redox mechanism. In conclusion, GKT136901 and GKT137831 might be promising clinical candidates, however their mechanism of action is not explained by NOX inhibition and previous reports using these compounds should be reconsidered in light of this fact.

Altogether our data on widely used inhibitors highlight the lack of reliable and specific NOX inhibitors as well as the problems of the systems used to identify NOX inhibitors. Other documented NOX inhibitors have not been included in this study, but are rarely used as they are either not commercially available (e.g. WO2016207785; WO2018203298), have not been through a full validation process [57] or present other pharmacological properties potentially obscuring the NOX inhibitory effect [58]. GSK2795039 stands out as a competitive NOX2 inhibitor as off target effects, specificity, mechanism of action, bioavailability and *in vivo* efficacy were carefully addressed [8]. GLX7013114 was recently described as NOX4-specific; unfortunately the structure of this compound has not yet been disclosed [21]. Except for DPI, which is not useful *in vivo*, currently available reported NOX inhibitors mostly act in fact as redox modifiers. In spite of potential therapeutic benefit, their use is not more useful than general redox-active agents, such as N-acetyl-cysteine to decipher the role of NOX *in vivo*. Screening of new chemical entities and systematic evaluation of their effects in multiple test systems is required to identify the novel specific molecules useful to address the impact of NOX isoforms in disease.

## Author contributions

FA, AF, DR, TS, ML, GM, ZM and VJ performed experiments; UGK provided DUOX1 and DUOX2 antibodies, PJD provided NOX4 antibody, JD provided NOX1 antibody; PJD, UGK and RS edited the text. AF, FA and VJ wrote the manuscript and prepared the figures. VJ, KHK and RS designed the experiments.

## Conflicts of interest

VJ and KHK hold shares of Genkyotex SA, a company aiming at developing NOX inhibitors. All authors declare no conflicts of interests.

## References

[1] Bedard K., Krause K.H. (2007). The NOX family of ROS-generating NADPH oxidases: physiology and pathophysiology. Physiol. Rev..

[2] van der Weyden L., Arends M.J., Campbell A.D., Bald T., Wardle-Jones H., Griggs N., Velasco-Herrera M.D., Tuting T., Sansom O.J., Karp N.A., Clare S., Gleeson D., Ryder E., Galli A., Tuck E., Cambridge E.L., Voet T., Macaulay I.C., Wong K., Sanger Mouse Genetics P., Spiegel S., Speak A.O., Adams D.J. (2017). Genome-wide in vivo screen identifies novel host regulators of metastatic colonization. Nature.

[3] Casas A.I., Geuss E., Kleikers P.W.M., Mencl S., Herrmann A.M., Buendia I., Egea J., Meuth S.G., Lopez M.G., Kleinschnitz C., Schmidt H. (2017). NOX4-dependent neuronal autotoxicity and BBB breakdown explain the superior sensitivity of the brain to ischemic damage. Proc. Natl. Acad. Sci. U. S. A..

[4] Di Marco E., Gray S.P., Kennedy K., Szyndralewiez C., Lyle A.N., Lassegue B., Griendling K.K., Cooper M.E., Schmidt H., Jandeleit-Dahm K.A.M. (2016). NOX4-derived reactive oxygen species limit fibrosis and inhibit proliferation of vascular smooth muscle cells in diabetic atherosclerosis. Free Radic. Biol. Med..

[5] Altenhofer S., Radermacher K.A., Kleikers P.W., Wingler K., Schmidt H.H. (2015). Evolution of NADPH oxidase inhibitors: selectivity and mechanisms for target engagement. Antioxidants Redox Signal..

[6] Jaquet V., Scapozza L., Clark R.A., Krause K.H., Lambeth J.D. (2009). Small-molecule NOX inhibitors: ROS-generating NADPH oxidases as therapeutic targets. Antioxidants Redox Signal..

[7] Seredenina T., Chiriano G., Filippova A., Nayernia Z., Mahiout Z., Fioraso-Cartier L., Plastre O., Scapozza L., Krause K.H., Jaquet V. (2015). A subset of N-substituted phenothiazines inhibits NADPH oxidases. Free Radic. Biol. Med..

[8] Hirano K., Chen W.S., Chueng A.L., Dunne A.A., Seredenina T., Filippova A., Ramachandran S., Bridges A., Chaudry L., Pettman G., Allan C., Duncan S., Lee K.C., Lim J., Ma M.T., Ong A.B., Ye N.Y., Nasir S., Mulyanidewi S., Aw C.C., Oon P.P., Liao S., Li D., Johns D.G., Miller N.D., Davies C.H., Browne E.R., Matsuoka Y., Chen D.W., Jaquet V., Rutter A.R. (2015). Discovery of GSK2795039, a novel small molecule NADPH oxidase 2 inhibitor. Antioxidants Redox Signal..

[9] Zielonka J., Cheng G., Zielonka M., Ganesh T., Sun A., Joseph J., Michalski R., O'Brien W.J., Lambeth J.D., Kalyanaraman B. (2014). High-throughput assays for superoxide and hydrogen peroxide: design of a screening workflow to identify inhibitors of NADPH oxidases. J. Biol. Chem..

[10] Cross A.R., Jones O.T. (1986). The effect of the inhibitor diphenylene iodonium on the superoxide-generating system of neutrophils. Specific labelling of a component polypeptide of the oxidase. Biochem. J..

[11] Banfi B., Malgrange B., Knisz J., Steger K., Dubois-Dauphin M., Krause K.H. (2004). NOX3, a superoxide-generating NADPH oxidase of the inner ear. J. Biol. Chem..

[12] Ueno N., Takeya R., Miyano K., Kikuchi H., Sumimoto H. (2005). The NADPH oxidase Nox3 constitutively produces superoxide in a p22phox-dependent manner: its regulation by oxidase organizers and activators. J. Biol. Chem..

[13] Nauseef W.M. (2007). Isolation of human neutrophils from venous blood. Methods Mol. Biol..

[14] Antony S., Wu Y., Hewitt S.M., Anver M.R., Butcher D., Jiang G., Meitzler J.L., Liu H., Juhasz A., Lu J., Roy K.K., Doroshow J.H. (2013). Characterization of NADPH oxidase 5 expression in human tumors and tumor cell lines with a novel mouse monoclonal antibody. Free Radic. Biol. Med..

[15] Verhoeven A.J., Bolscher B.G., Meerhof L.J., van Zwieten R., Keijer J., Weening R.S., Roos D. (1989). Characterization of two monoclonal antibodies against cytochrome b558 of human neutrophils. Blood.

[16] Meitzler J.L., Makhlouf H.R., Antony S., Wu Y., Butcher D., Jiang G., Juhasz A., Lu J., Dahan I., Jansen-Durr P., Pircher H., Shah A.M., Roy K., Doroshow J.H. (2017). Decoding NADPH oxidase 4 expression in human tumors. Redox Biol..

[17] Serrander L., Jaquet V., Bedard K., Plastre O., Hartley O., Arnaudeau S., Demaurex N., Schlegel W., Krause K.H. (2007). NOX5 is expressed at the plasma membrane and generates superoxide in response to protein kinase C activation. Biochimie.

[18] Luxen S., Belinsky S.A., Knaus U.G. (2008). Silencing of DUOX NADPH oxidases by promoter hypermethylation in lung cancer. Cancer Res..

[19] Maghzal G.J., Cergol K.M., Shengule S.R., Suarna C., Newington D., Kettle A.J., Payne R.J., Stocker R. (2014). Assessment of myeloperoxidase activity by the conversion of hydroethidine to 2-chloroethidium. J. Biol. Chem..

[20] Zhang K.M., Dou W., Li P.X., Shen R., Ru J.X., Liu W., Cui Y.M., Chen C.Y., Liu W.S., Bai D.C. (2015). A coumarin-based two-photon probe for hydrogen peroxide. Biosens. Bioelectron..

[21] Wang X., Elksnis A., Wikstrom P., Walum E., Welsh N., Carlsson P.O. (2018). The novel NADPH oxidase 4 selective inhibitor GLX7013114 counteracts human islet cell death in vitro. PLoS One.

[22] Miyano K., Sumimoto H. (2014). N-Linked glycosylation of the superoxide-producing NADPH oxidase Nox1. Biochem. Biophys. Res. Commun..

[23] Petheo G.L., Orient A., Barath M., Kovacs I., Rethi B., Lanyi A., Rajki A., Rajnavolgyi E., Geiszt M. (2010). Molecular and functional characterization of Hv1 proton channel in human granulocytes. PLoS One.

[24] Anilkumar N., San Jose G., Sawyer I., Santos C.X., Sand C., Brewer A.C., Warren D., Shah A.M. (2013). A 28-kDa splice variant of NADPH oxidase-4 is nuclear-localized and involved in redox signaling in vascular cells. Arterioscler. Thromb. Vasc. Biol..

[25] Luxen S., Noack D., Frausto M., Davanture S., Torbett B.E., Knaus U.G. (2009). Heterodimerization controls localization of Duox-DuoxA NADPH oxidases in airway cells. J. Cell Sci..

[26] Zielonka J., Lambeth J.D., Kalyanaraman B. (2013). On the use of L-012, a luminol-based chemiluminescent probe, for detecting superoxide and identifying inhibitors of NADPH oxidase: a reevaluation. Free Radic. Biol. Med..

[27] Ueyama T., Sakuma M., Ninoyu Y., Hamada T., Dupuy C., Geiszt M., Leto T.L., Saito N. (2015). The extracellular A-loop of dual oxidases affects the specificity of reactive oxygen species release. J. Biol. Chem..

[28] Nisimoto Y., Diebold B.A., Cosentino-Gomes D., Lambeth J.D. (2014). Nox4: a hydrogen peroxide-generating oxygen sensor. Biochemistry.

[29] Szilagyi J.T., Mishin V., Heck D.E., Jan Y.H., Aleksunes L.M., Richardson J.R., Heindel N.D., Laskin D.L., Laskin J.D. (2016). Selective targeting of heme protein in cytochrome P450 and nitric oxide synthase by diphenyleneiodonium. Toxicol. Sci..

[30] Stuehr D.J., Fasehun O.A., Kwon N.S., Gross S.S., Gonzalez J.A., Levi R., Nathan C.F. (1991). Inhibition of macrophage and endothelial cell nitric oxide synthase by diphenyleneiodonium and its analogs. FASEB J..

[31] Holland P.C., Sherrat H.S. (1971). Biochemical effects of hypoglycaemic compound diphenyleneiodonium in rat liver mitochondria: inhibition of adenosine triphosphate synthesis. Biochem. J..

[32] Gatley S.J., Martin J.L. (1979). Some aspects of the pharmacology of diphenyleneiodonium, a bivalent iodine compound. Xenobiotica.

[33] Jaquet V., Marcoux J., Forest E., Leidal K.G., McCormick S., Westermaier Y., Perozzo R., Plastre O., Fioraso-Cartier L., Diebold B., Scapozza L., Nauseef W.M., Fieschi F., Krause K.H., Bedard K. (2011). NADPH oxidase (NOX) isoforms are inhibited by celastrol with a dual mode of action. Br. J. Pharmacol..

[34] Noguchi N., Yoshida Y., Kaneda H., Yamamoto Y., Niki E. (1992). Action of ebselen as an antioxidant against lipid peroxidation. Biochem. Pharmacol..

[35] Smith S.M., Min J., Ganesh T., Diebold B., Kawahara T., Zhu Y., McCoy J., Sun A., Snyder J.P., Fu H., Du Y., Lewis I., Lambeth J.D. (2012). Ebselen and congeners inhibit NADPH oxidase 2-dependent superoxide generation by interrupting the binding of regulatory subunits. Chem. Biol..

[36] Hanavan P.D., Borges C.R., Katchman B.A., Faigel D.O., Ho T.H., Ma C.T., Sergienko E.A., Meurice N., Petit J.L., Lake D.F. (2015). Ebselen inhibits QSOX1 enzymatic activity and suppresses invasion of pancreatic and renal cancer cell lines. Oncotarget.

[37] Wang Y., Wallach J., Duane S., Wang Y., Wu J., Wang J., Adejare A., Ma H. (2017). Developing selective histone deacetylases (HDACs) inhibitors through ebselen and analogs. Drug Des. Dev. Ther..

[38] Kil J., Lobarinas E., Spankovich C., Griffiths S.K., Antonelli P.J., Lynch E.D., Le Prell C.G. (2017). Safety and efficacy of ebselen for the prevention of noise-induced hearing loss: a randomised, double-blind, placebo-controlled, phase 2 trial. Lancet.

[39] Beckman J.A., Goldfine A.B., Leopold J.A., Creager M.A. (2016). Ebselen does not improve oxidative stress and vascular function in patients with diabetes: a randomized, crossover trial. Am. J. Physiol. Heart Circ. Physiol..

[40] ten Freyhaus H., Huntgeburth M., Wingler K., Schnitker J., Baumer A.T., Vantler M., Bekhite M.M., Wartenberg M., Sauer H., Rosenkranz S. (2006). Novel Nox inhibitor VAS2870 attenuates PDGF-dependent smooth muscle cell chemotaxis, but not proliferation. Cardiovasc. Res..

[41] Gatto G.J., Ao Z., Kearse M.G., Zhou M., Morales C.R., Daniels E., Bradley B.T., Goserud M.T., Goodman K.B., Douglas S.A., Harpel M.R., Johns D.G. (2013). NADPH oxidase-dependent and -independent mechanisms of reported inhibitors of reactive oxygen generation. J. Enzym. Inhib. Med. Chem..

[42] Sun Q.A., Hess D.T., Wang B., Miyagi M., Stamler J.S. (2012). Off-target thiol alkylation by the NADPH oxidase inhibitor 3-benzyl-7-(2-benzoxazolyl)thio-1,2,3-triazolo[4,5-d]pyrimidine (VAS2870). Free Radic. Biol. Med..

[43] Fradin T., Bechor E., Berdichevsky Y., Dahan I., Pick E. (2018). Binding of p67(phox) to Nox2 is stabilized by disulfide bonds between cysteines in the (369) Cys-Gly-Cys(371) triad in Nox2 and in p67(phox). J. Leukoc. Biol..

[44] Petrushanko I.Y., Lobachev V.M., Kononikhin A.S., Makarov A.A., Devred F., Kovacic H., Kubatiev A.A., Tsvetkov P.O. (2016). Oxidation of capital ES, cyrillicsmall a, cyrillic2+-binding domain of NADPH oxidase 5 (NOX5): toward understanding the mechanism of inactivation of NOX5 by ROS. PLoS One.

[45] Meitzler J.L., Hinde S., Banfi B., Nauseef W.M., Ortiz de Montellano P.R. (2013). Conserved cysteine residues provide a protein-protein interaction surface in dual oxidase (DUOX) proteins. J. Biol. Chem..

[46] Chandasana H., Chhonker Y.S., Bala V., Prasad Y.D., Chaitanya T.K., Sharma V.L., Bhatta R.S. (2015). Pharmacokinetic, bioavailability, metabolism and plasma protein binding evaluation of NADPH-oxidase inhibitor apocynin using LC-MS/MS. J. Chromatogr. B Analyt. Technol. Biomed. Life. Sci..

[47] Virdis A., Gesi M., Taddei S. (2016). Impact of apocynin on vascular disease in hypertension. Vasc. Pharmacol..

[48] Stefanska J., Sarniak A., Wlodarczyk A., Sokolowska M., Pniewska E., Doniec Z., Nowak D., Pawliczak R. (2012). Apocynin reduces reactive oxygen species concentrations in exhaled breath condensate in asthmatics. Exp. Lung Res..

[49] Stolk J., Hiltermann T.J., Dijkman J.H., Verhoeven A.J. (1994). Characteristics of the inhibition of NADPH oxidase activation in neutrophils by apocynin, a methoxy-substituted catechol. Am. J. Respir. Cell Mol. Biol..

[50] Ximenes V.F., Kanegae M.P., Rissato S.R., Galhiane M.S. (2007). The oxidation of apocynin catalyzed by myeloperoxidase: proposal for NADPH oxidase inhibition. Arch. Biochem. Biophys..

[51] Van den Worm E., Beukelman C.J., Van den Berg A.J., Kroes B.H., Labadie R.P., Van Dijk H. (2001). Effects of methoxylation of apocynin and analogs on the inhibition of reactive oxygen species production by stimulated human neutrophils. Eur. J. Pharmacol..

[52] Ismail H.M., Scapozza L., Ruegg U.T., Dorchies O.M. (2014). Diapocynin, a dimer of the NADPH oxidase inhibitor apocynin, reduces ROS production and prevents force loss in eccentrically contracting dystrophic muscle. PLoS One.

[53] Ghosh A., Kanthasamy A., Joseph J., Anantharam V., Srivastava P., Dranka B.P., Kalyanaraman B., Kanthasamy A.G. (2012). Anti-inflammatory and neuroprotective effects of an orally active apocynin derivative in pre-clinical models of Parkinson's disease. J. Neuroinflammation.

[54] Teixeira G., Szyndralewiez C., Molango S., Carnesecchi S., Heitz F., Wiesel P., Wood J.M. (2017). Therapeutic potential of NADPH oxidase 1/4 inhibitors. Br. J. Pharmacol..

[55] Holdgate G.A., Meek T.D., Grimley R.L. (2018). Mechanistic enzymology in drug discovery: a fresh perspective. Nat. Rev. Drug Discov..

[56] Laleu B., Gaggini F., Orchard M., Fioraso-Cartier L., Cagnon L., Houngninou-Molango S., Gradia A., Duboux G., Merlot C., Heitz F., Szyndralewiez C., Page P. (2010). First in class, potent, and orally bioavailable NADPH oxidase isoform 4 (Nox4) inhibitors for the treatment of idiopathic pulmonary fibrosis. J. Med. Chem..

[57] Joo J.H., Huh J.E., Lee J.H., Park D.R., Lee Y., Lee S.G., Choi S., Lee H.J., Song S.W., Jeong Y., Goo J.I., Choi Y., Baek H.K., Yi S.S., Park S.J., Lee J.E., Ku S.K., Lee W.J., Lee K.I., Lee S.Y., Bae Y.S. (2016). A novel pyrazole derivative protects from ovariectomy-induced osteoporosis through the inhibition of NADPH oxidase. Sci. Rep..

[58] Seredenina T., Nayernia Z., Sorce S., Maghzal G.J., Filippova A., Ling S.C., Basset O., Plastre O., Daali Y., Rushing E.J., Giordana M.T., Cleveland D.W., Aguzzi A., Stocker R., Krause K.H., Jaquet V. (2016). Evaluation of NADPH oxidases as drug targets in a mouse model of familial amyotrophic lateral sclerosis. Free Radic. Biol. Med..

